# Caffeic Acid/Eu(III) Complexes: Solution Equilibrium Studies, Structure Characterization and Biological Activity

**DOI:** 10.3390/ijms23020888

**Published:** 2022-01-14

**Authors:** Żaneta Arciszewska, Sofia Gama, Monika Kalinowska, Grzegorz Świderski, Renata Świsłocka, Ewelina Gołębiewska, Monika Naumowicz, Mateusz Worobiczuk, Adam Cudowski, Anna Pietryczuk, Concetta De Stefano, Demetrio Milea, Włodzimierz Lewandowski, Beata Godlewska-Żyłkiewicz

**Affiliations:** 1 Department of Analytical and Inorganic Chemistry, Faculty of Chemistry, University of Bialystok, K. Ciołkowskiego 1K, 15-245 Białystok, Poland; z.arciszewska@uwb.edu.pl; 2 Department of Chemistry, Biology and Biotechnology, Bialystok University of Technology, Wiejska 45E, 15-351 Białystok, Poland; m.kalinowska@pb.edu.pl (M.K.); g.swiderski@pb.edu.pl (G.Ś.); r.swislocka@pb.edu.pl (R.Ś.); e.golebiewska@pb.edu.pl (E.G.); 3 Department of Physical Chemistry, Faculty of Chemistry, University of Bialystok, K. Ciołkowskiego 1K, 15-245 Białystok, Poland; monikan@uwb.edu.pl (M.N.); mworobiczuk@gmail.com (M.W.); 4 Department of Water Ecology, Faculty of Biology, University of Bialystok, K. Ciołkowskiego 1J, 15-245 Białystok, Poland; cudad@uwb.edu.pl (A.C.); annapiet@uwb.edu.pl (A.P.); 5 Dipartimento di Scienze Chimiche, Biologiche, Farmaceutiche ed Ambientali, CHIBIOFARAM, Università degli Studi di Messina, V.le F. Stagno d’Alcontres, 31, 98166 Messina, Italy; cdestefano@unime.it (C.D.S.); dmilea@unime.it (D.M.); 6 Prof Waclaw Dabrowski Institute of Agricultural and Food Biotechnology—State Research Institute, 36 Rakowiecka St., 02-532 Warsaw, Poland; w.lewandowski@pb.edu.pl

**Keywords:** antimicrobial activity, antioxidant activity, chemical speciation, lanthanides, metal complexes, microelectrophoretic mobility, polyphenols, sequestering ability, solution equilibria

## Abstract

Caffeic acid (CFA) is one of the various natural antioxidants and chemoprotective agents occurring in the human diet. In addition, its metal complexes play fundamental roles in biological systems. Nevertheless, research on the properties of CFA with lanthanide metals is very scarce, and little to no chemical or biological information is known about these particular systems. Most of their properties, including their biological activity and environmental impact, strictly depend on their structure, stability, and solution behaviour. In this work, a multi-analytical-technique approach was used to study these relationships for the Eu(III)/CFA complex. The synthesized metal complex was studied by FT-IR, FT-Raman, elemental, and thermal (TGA) analysis. In order to examine the chemical speciation of the Eu(III)/CFA system in an aqueous solution, several independent potentiometric and spectrophotometric UV-Vis titrations were performed at different M:L (metal:ligand) and pH ratios. The general molecular formula of the synthesized metal complex in the solid state was [Eu(CFA)_3_(H_2_O)_3_]∙2H_2_O (M:L ratio 1:3), while in aqueous solution the 1:1 species were observed at the optimum pH of 6 ≤ pH ≤ 10, ([Eu(CFA)] and [Eu(CFA)(OH)]^−^). These results were confirmed by ^1^H-NMR experiments and electrospray-ionization mass spectrometry (ESI-MS). To evaluate the interaction of Eu(III)/CFA and CFA alone with cell membranes, electrophoretic mobility assays were used. Various antioxidant tests have shown that Eu(III)/CFA exhibits lower antioxidant activity than the free CFA ligand. In addition, the antimicrobial properties of Eu(III)/CFA and CFA against *Escherichia coli*, *Bacillus subtilis* and *Candida albicans* were investigated by evaluation of the minimum inhibitory concentration (MIC). Eu(III)/CFA shows higher antibacterial activity against bacteria compared to CFA, which can be explained by the highly probable increased lipophilicity of the Eu(III) complex.

## 1. Introduction

Natural carboxylic acids occur widely in nature. Among them, polyphenols are considered to be the most important agents responsible for many biological functions. Plant phenolics include simple phenols, phenolic acids, flavonoids, coumarins, stilbenes, hydrolysable and condensed tannins, lignans, and lignins. They can be found in the edible parts of many plants such as fruits, seeds, leaves, stems, and roots. Their occurrence, distribution in plants, structure, biochemical activity and other unique properties have been extensively studied in the field of bioinorganic, food and pharmaceutical chemistry, due to their various biological and pharmacological activities, including antioxidant, antimicrobial and anticancer activities [1].

**Scheme 1:**
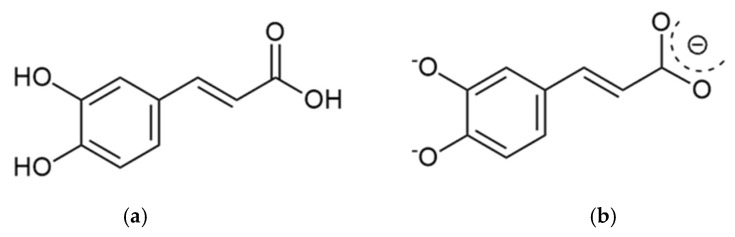
Chemical structure of CFA in its fully protonated (CFA, LH_3_) and deprotonated (L^3−^) forms: (**a**) caffeic acid (CFA, LH_3_); (**b**) caffeinate (L^3−^).

Polyphenolic acids can be divided into two major groups: hydroxybenzoic and hydroxycinnamic acids. All compounds contain one or more hydroxyl groups attached directly to the aromatic ring. The caffeic acid (3-(3,4-Dihydroxyphenyl)-2-propenoic acid, Scheme 1a), from now on designated as CFA (in its fully deprotonated form of caffeinate it will be denoted as L^3−^, Scheme 1b), is one of the most abundant hydroxycinnamic acids, with a very well demonstrated antibacterial activity, and is described in numerous publications [1,2,3,4,5,6,7]. The observed biological activity seems to result from the ability of CFA to damage the cell membrane integrity and/or change its permeability, to inhibit some particular enzymatic activity, to trigger protein damage, or to change DNA structure [7].

In spite of the important role of this molecule in the environmental and biological fields, including human nutrition, very little is known about the structure and properties of CFA complexes with metal ions, especially with lanthanides. The synthesis of metal complexes and their spectroscopic studies are described in some cases; however information on their biological activity is scarce, although the chemoprotective activity of this acid is often related to the coordination or reduction of transition metals and the formation of adducts [1,8]. Studies of the structure of CFA complexes with a series of cations, namely Li^+^, Na^+^, K^+^, Rb^+^, and Cs^+^, performed in the solid state, show that considered metals affect the molecular structure and electronic charge distribution of the ligand; this changes their biological action, namely their antioxidant activity [9,10]. In an aqueous solution, the coordination of metal cations seems to occur preferentially by the hydroxyl groups. Cornard et al. [11,12] described the formation of Al(III)-CFA complexes and, using UV-Vis and synchronous fluorescence spectroscopies, demonstrated that, for example, only the catechol moiety is involved in the formation of 1:2 and 1:3 complexes at pH = 6.5 [12]. This was also observed for Cr(III), as the binding of ions takes place through the two phenolic groups of the CFA [13]. Nevertheless, the formation of CFA-Pb(II) metal complexes in aqueous solutions seems to occur directly via carboxylic group [14]. Many other studies on the interaction of CFA toward different metal cations are reported [15,16] but, to our knowledge, no work was reported concerning the study of the complexes formed for CFA/lanthanide(III) systems.

Lanthanides are well-known for their particular chemical properties that lead to their application in several fields, from electronics and physics to medical applications (therapeutics and/or diagnosis) [17]. However, the biological role of lanthanides and their impact on living organisms is still poorly understood, although it is well-known that they interact with several biologically relevant ligands [18,19,20]. Several Ln(III) complexes with organic ligands show interesting pharmacological properties. For example, Kostova and co-workers studied the molecular structure and spectroscopic properties of various derivatives of coumarin and orotic acid (vitamin B13) and their complexes with La(III), Ce(III), Nd(III), Dy(III), and Sm(III), as well as the antioxidant activity of the formed complexes [21,22,23,24]. Other authors documented higher antibacterial activity of Ln(III) (Dy, Sm, Pr, Nd, La, Er, and Gd) complexes with tetradentate Schiff bases when compared to free ligands [25,26]. According to the recent review of Cota et al. [27], the antimicrobial activity of ligands is enhanced by the complexation with lanthanide ions, mainly due to the increase in the lipophilicity of the metal complex vs. the free ligand. This helps the penetration through the lipid membranes, disturbing the functioning of the bacterial/fungal cell. Moreover, the anticancer activity of lanthanides was also evaluated and several Eu(III) complexes were synthesized and tested, showing some promising results as new anticancer agents [28,29,30].

As part of a wider topic which aims to study the relationship between the molecular structure and the biological activity (microbial, cytostatic and antioxidant) of selected phenolic acids and their metal complexes [31,32,33,34], we present herein the study of the interaction of CFA towards Eu(III), and how the complexation influences the antimicrobial and the antioxidative properties of CFA. On a global perspective, we intend to contribute to a better understanding of how coordination with selected metals affects the molecular structure and distribution of the electronic charge of ligands, changing CFA biological activity, including their antioxidant properties. To answer this, we need to exploit several topics: (1) which binding groups of CFA are involved in the coordination of Eu(III), and how this affects the biological activity of the metal complex when compared with the free ligand; (2) how the extension of the conjugated system of double bonds (responsible for the delocalization of the electronic charge) will translate into biological properties, by comparing different benzoic acid derivatives (with a shorter conjugated double bond system) with cinnamic acid derivatives (with a longer double conjugated bond system); (3) which metal parameters have the greatest impact on the increase in the antioxidant properties of ligands due to metal complexation.

Due to the complexity of the systems under study, only a multi-analytical-technique approach can provide sufficient knowledge for answering all of these questions; these can be answered by means of investigation of solution equilibria [35], the correct characterization of the synthesized compounds [36,37], and the assessment of their biological activity [38]. In our previous works, the above-mentioned effects were mainly examined in the solid phase [32,34,39,40]. Currently, this paper is also devoted to the study of the chemical equilibria, speciation and sequestering ability of CFA in aqueous solution, as they are of paramount relevance to the evaluation of the biological activity of metal compounds; this is due to modulation of the conditions existing in the media used to perform some of the assays, and at a later stage, inside living organisms. For this reason the chemical equilibria of the Eu(III)/CFA system in an aqueous solution were studied using a multi-technique approach, where potentiometry and UV-Vis spectroscopy measurements were complemented by ^1^H-NMR experiments and electrospray-ionization mass spectrometry (ESI-MS) to support the existence of EuL species. The Eu(III)-CFA metal complex was also synthesized and characterized using elemental and thermal analyses, as well as Raman and FT-IR spectroscopy. Moreover, the antioxidant and antimicrobial activity of Eu(III)-CFA was evaluated and compared with CFA action. This was achieved by means of several antioxidant assays based on different mechanisms of action, namely DPPH• (2,2-diphenyl-1-picrylhydrazyl) radical, ABTS•+ (2,2-azino-bis(3-ethylbenzothiazoline-6-sulfonic acid), FRAP (ferric reducing antioxidant power), CUPRAC (cupric reducing antioxidant power), and lipid peroxidation assays. The antimicrobial activity against *Escherichia coli* (Gram(−) bacteria), *Bacillus subtilis* (Gram(+) bacteria) and *Candida albicans* (fungi) was also tested, while electrophoretic mobility assays were used to evaluate the interaction of CFA and CFA/Eu(III) with the cell membranes of the studied microorganisms.

## 2. Results and Discussion

### 2.1. Molecular Structure of [Eu(CFA)_3_]

**Figure 1:**
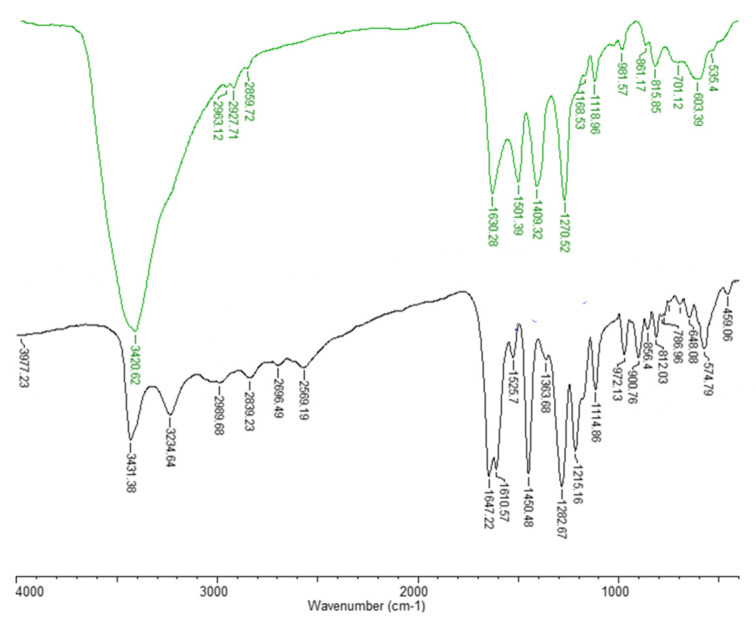
FT-IR spectra (registered in KBr pellets) for CFA (black) and Eu(III)-caffeinate (green).

The structure of the europium complex was described based on the results of elemental analysis and TGA, combined with spectroscopic studies. For that purpose, FT-IR and FT Raman spectra of CFA and Eu-caffeinate were recorded. The spectroscopic data are presented in Table 1, and the recorded FT-IR spectra are depicted in Figure 1. The vibration bands of the aromatic system have been numbered according to Varsanyi’s classification [41]. The spectra of CFA show characteristic bands derived from the stretching vibrations of the carbonyl group ν_C=O_, present at the values of 1647 cm^−1^ (IR_KBr_), 1634 cm^−1^ (IR_ATR_) and 1641 cm^−1^ (Raman). This band overlaps with the band of the stretching vibrations of the ν_(CH)C=C_ group. The CFA spectra also show characteristic bands derived from the vibrations of the hydroxyl groups attached to the aromatic ring, at ca. 1280 cm^−1^, being the stretching vibrations of the hydroxyl group (ν_O–H_) present, observed at 3431 cm^−1^ and 3235 cm^−1^. When comparing the CFA spectra with those obtained for Eu-caffeinate, the first observation that can be made is that the bands associated with the stretching vibrations of the aromatic ring hydroxyl groups in the europium complex have a significantly reduced intensity compared to these bands in the CFA spectrum. It was also observed that some of the bands associated with the bending vibration of the hydroxyl groups disappeared in the spectra of the europium complex, or were of reduced intensity. This could be the result of the involvement of the hydroxyl groups in the coordination of the metal cation. Furthermore, new characteristic bands related to the vibration of the carboxylate anion appear on the spectrum of the analysed europium complex. These are the bands of symmetric stretching vibrations ν_s_COO^−^ present at 1409 cm^−1^ (IR_KBr_, IR_ATR_) in the europium complex, and bands of symmetric stretching vibrations ν_as_COO^−^ presented at 1501 cm^−1^ (IR_KBr_) and 1499 cm^−1^ (IR_ATR_). In addition, the spectra of the tested metal complex show bands associated with vibrations bending in the plane — β_s_COO^−^ (symmetrical) and β_as_COO^−^ (asymmetrical) — and bending out of the plane (γCOO^−^) (See Table 1). A small shift to lower wavelength is also observed for the bands related to the stretching vibrations of the carbonyl group (ν_C=O_), from 1647 cm^−1^ to 1630 cm^−1^ (IR_KBr_), and 1634 cm^−1^ to 1629 cm^−1^ (IR_ATR_). This may indicate the occurrence of some interaction of the metal cation with the ligand, this time through the carboxyl group.

In an effort to obtain a better insight on the possible structure of the Eu-caffeinate complex, we analysed, in more detail, the changes in the aromatic system of CFA resulting from the coordination to the metal cation. In general, lanthanides, similarly to 3d-transition metals, stabilize the electronic system of the aromatic ring in ligands as benzoates, salicylates, and 3-pyridine carboxylates, resulting from the coordination to the carboxylic group of the benzoate ring [42,43]. This results in substantial changes in the correspondent IR spectra, usually characterized by an increase in the number and intensity of the aromatic system bands, as well as a shift on their wavenumber values, when compared with the free ligand spectra. On the contrary, when the aromatic system is destabilized by the coordination of, for example, alkaline metals, we observe a decrease in intensity in the spectra and a decrease in the wavenumber values of the bands of the aromatic system [42,43]. In the particular case of the Eu-caffeinate spectrum, even though we observed a small increase in the wavenumbers of some bands of the aromatic system (e.g., 6b, 17b and 18b, designated according to Varsányi’s classification [41]), most of them disappear, or their wavenumber values are shifted towards lower values in the spectrum of Eu(III)-caffeinate when compared to the spectrum of CFA. This may indicate that, in this particular case, the coordination of Eu(III) has a destabilizing effect on the aromatic system of CFA, possibly the result of a not so relevant interaction between the carboxylic group and the metal cation. Nevertheless, if it happens, we can predict the type of metal–ligand coordination in the studied Eu(III) complex, based on the criteria described by Nakamoto and McCarthy [44]. According to them, when the difference between the wavenumbers of asymmetric and symmetric vibrations of the COO^−^ group in the IR spectrum of the studied complex (Δν(COO^−^) = ν_as_(COO^−^) − ν_s_(COO^−^)) is much smaller than the difference between the wavenumbers of the same bands for sodium salt, then there is presumably a bidentate chelating type of coordination. A Δν(COO^−^)_complex_ equal to or lower than Δν(COO^−^)_Na salt_ may be indicative of a bidentate bridging coordination type. Finally, when Δν(COO^−^)_complex_ is much higher than Δν(COO^−^)_Na salt_, we may be in the presence of a monodentate coordination type. As such, in the case of the metal complex of Eu(III) with CFA, the analysis of the asymmetric and symmetric stretching vibrations of the carboxylate anion, in relation to the analogous vibrations in the sodium salt (Figure S1), indicates a bidentate coordination type for carboxylic moiety chelation (Δν(COO^−^)_complex_ = 92 cm^−1^ << Δν(COO^−^)_Na salt_ = 147 cm^−1^). Nevertheless, it is worth mentioning that, if the coordination of CFA molecules to the central europium ion occurs through the hydroxyl groups of the aromatic ring, a carboxylate anion is formed, in which the carbon–oxygen bonds are aligned. Hence, the position of the asymmetric and symmetric stretching bands of the carboxylate anion may be similar to those of a CFA molecule that coordinates Eu(III) via the carboxyl group, also resulting in a small Δν(COO^−^)_complex_.

For comparative purposes, Eu(III) complexes of cinnamic and *p*-coumaric acids were also synthesized, and the metal complexes’ spectroscopic data, as well as those of their corresponding ligands are reported in Table 2.

Cinnamic acid does not contain hydroxyl groups in the aromatic ring, while *p*-coumaric acid has one hydroxyl group in the para-position of the aromatic ring. Based on the chemical structure of the ligands and, once again, following Nakamoto’s criteria [44], we could predict the type of coordination of Eu(III) by *p*-coumaric acid and cinnamic acid. The bands resulting from the vibrations of the hydroxyl group of the aromatic *p*-coumaric acid system on the spectrum of the complex do not undergo significant changes in the metal complex; this proves that it is not involved in the metal coordination. Furthermore, taking into account Δν(COO^−^) = ν_as_(COO^−^) − ν_s_(COO^−^) differences for the sodium salt of cinnamic acid [45] and the europium complex, it can be assumed that there is a double-bridge-chelating coordination between the central ion and the ligand ((Δν(COO^−^)_complex_ = 124 = Δν(COO^−^)_Na salt_ = 124 [45]. In the case of the europium complex with *p*-coumaric acid, (Δν(COO^−^)_complex_ = 150 > Δν(COO^−^)_Na salt_ = 135 [46], and so the metal–ligand coordination by the carboxylic group should be monodentate. For both of these acids, metal complexation occurs favourably via the carboxyl group.

**Figure 2:**
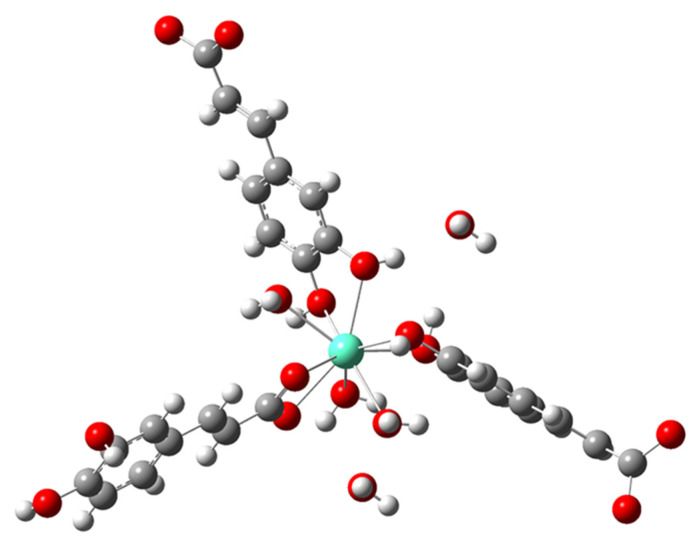
Proposed structure for the synthesized Eu(III)-caffeinate metal complex.

Therefore, we can try to propose a structure for the synthesized Eu(III)-caffeinate, also having as reference the various examples reported of metal complexes with hydroxybenzoic acids. In particular, in the metal complex of Eu(III) with gallic acid, described by Lin et al. [47], both the hydroxyl groups and the carboxyl group of gallic acid are involved in the metal–ligand coordination, but lead to the formation of an interesting 3D metal–organic framework. Another example is the study described by Dan et al. [48] of strontium complexes with dihydroxybenzoic acids. In this case, the complexation of strontium occurs through the participation of hydroxyl groups attached to the aromatic ring. The elemental analysis (EA) of the isolated solid indicated the occurrence of a total of five molecules of water per molecule of metal complex. This result was confirmed by thermal gravimetric analysis (TGA). During TGA two different steps of weight loss were observed: first at 40–110 °C, which we attributed to the thermal dehydration and loss of 2 water molecules (exp 6.32%, calc 5.75%), followed by a second one at 120–220 °C, corresponding to 3 water molecules coordinated to the metallic centre (exp 7.53%, calc 7.48%). On the basis of all of these considerations, we can propose the following structure for the Eu(III)-caffeinate complex (Figure 2), corresponding to the molecular formula [Eu(CFA)_3_(H_2_O)_3_]·2H_2_O.

### 2.2. Solution Equilibrium Studies

#### 2.2.1. Acid–Base Properties of CFA and Eu^3+^

The acid–base properties of CFA were studied and the protonation constants were determined experimentally by potentiometric titrations under the defined conditions of temperature, medium, and ionic strength (i.e., *T* = 298.15 ± 0.1 K and *I* = 0.2 mol·dm^−3^ in KCl_(aq)_). CFA has three functional groups that can be involved in various protonation/deprotonation equilibria, namely one carboxylic and two hydroxyl groups (forming a catecholate unit). In the pH range investigated (2 < pH < 12), three protonation steps can be observed, and the corresponding protonation constants are presented in Table 3.

**Figure 3:**
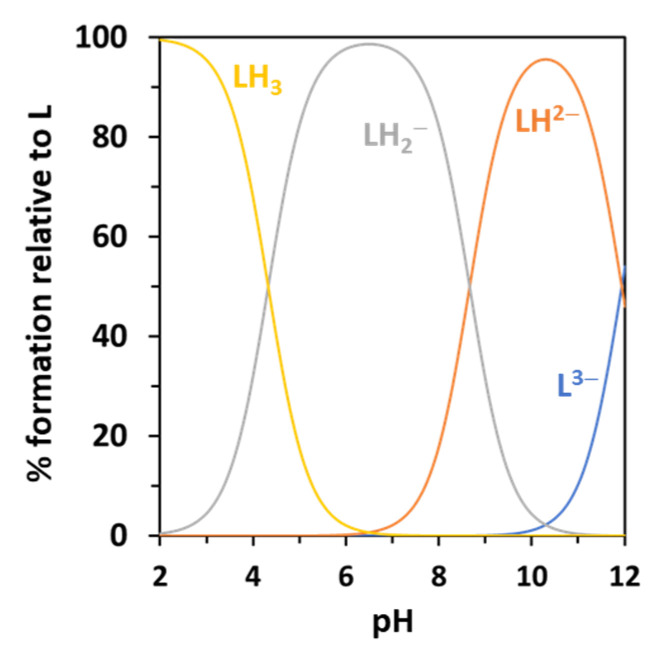
Distribution diagram of caffeinate species as a function of pH in KCl_(aq)_ at *I* = 0.2 mol·dm^−3^ and *T* = 298.15 K. *c*_L_ =1 mmol·dm^−3^.

Considering CFA’s molecular structure and based on several reported studies [15,49] the hydroxyl groups should be the first to be protonated (log *K* = 11.93 ± 0.02 and 8.66 ± 0.08), being the carboxylic group expected to be protonated as last, with an observed log *K* value of 4.33 ± 0.01, in the experimental conditions adopted. The obtained values are in full agreement with those reported in the literature by Bizri et al. [49] (log *K_011_* = 11.80, log *K_012_* = 8.66, log *K_013_* = 4.45). Based on the determined protonation constants, the distribution diagram of CFA species in different protonation states can be drawn, and it is presented in Figure 3.

The fully protonated species, [LH_3_], is the major species at pH < 4, when the deprotonation of the carboxylic acid starts, leading to the formation of [LH_2_]^−^ species, which is the major species at neutral pH. At pH > 8, the stepwise deprotonation of the catecholate unit starts, being the fully deprotonated species present only in very basic conditions (pH > 11).

**Figure 4:**
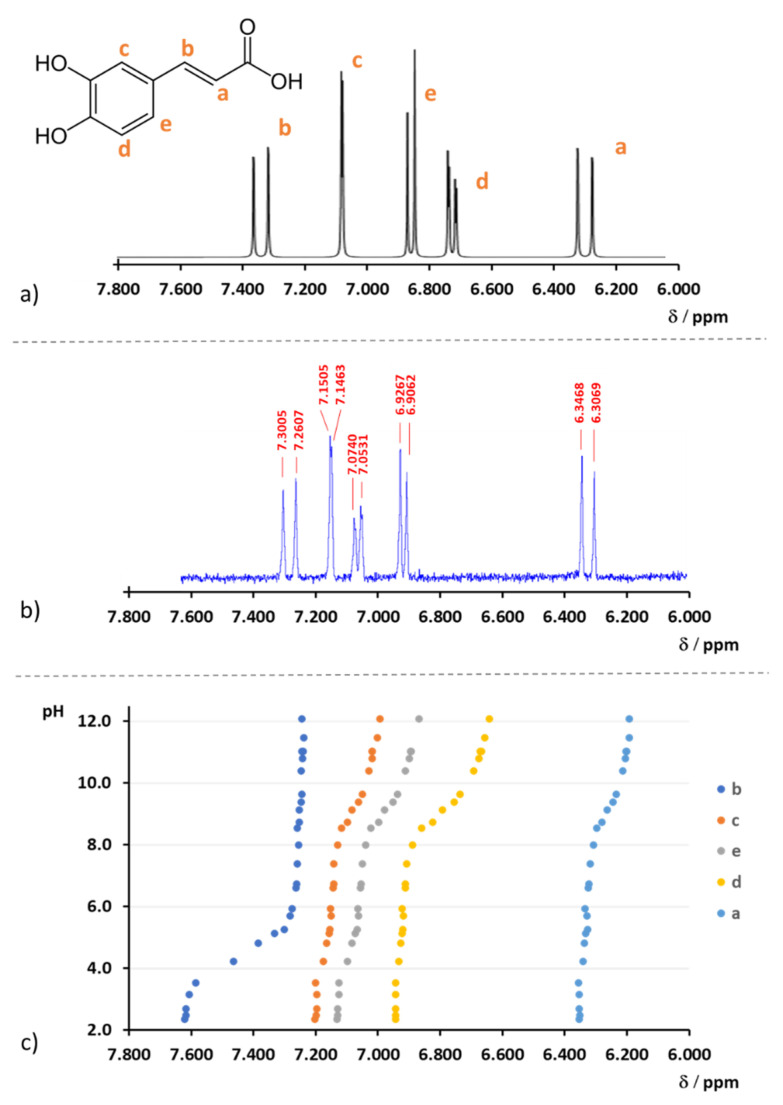
Representation of ^1^H-NMR titration: (**a**) ^1^H-NMR spectra of CFA calculated by ChemDraw software and corresponding peak attribution; (**b**) experimental ^1^H-NMR spectra of CFA (*c*_CFA_ = 10^−3^ mol·dm^−3^, pH = 5.7); (**c**) representation of the chemical shifts of each assigned peak obtained from experimental spectra recorded at different pH values.

Independent spectrophotometric titrations were also performed, and the fitting of the experimental data using the HypSpec2014 program [50] confirmed the protonation constants determined by potentiometric titrations (see Table 3). HypSpec2014 program fitting also allows the spectrophotometric characterization of the system calculating the molar absorptivity of each CFA species (Figure S2). These data (reported in Table S2) are in excellent agreement (practically coincident, within the uncertainty) with the literature [15]. The clear attribution of the protonation steps to the specific basic groups of CFA was achieved using ^1^H-NMR titrations. Several measurements were performed where the individual ^1^H-NMR spectra were recorded from CFA aqueous solutions at different pH values (see Figure S3). The individual chemical shift of each proton for each chemical species and their variations in the various protonation steps, graphically represented in Figure 4c (the experimental spectra can be seen in Figure S2 and the corresponding chemical shifts are reported in Table S1), provide support to the attribution of the protonation steps to specific basic groups. In particular, the variation of the individual chemical shift of H_b_ confirms the deprotonation of the carboxylic acid at pH ≈ 4 (with the calculated p*K*_a_ of 4.4 ± 0.1, see Table 3) and the catecholate deprotonation that occurs at pH > 8, evidenced by the change in the chemical shift of H_c_, H_d_ and H_e_. The obtained data (the chemical shift vs. pH) were fitted by HypNMR software [51] to determine the theoretical chemical shifts of each species (Table 4) as well as the protonation constants of CFA (Table 3). Data reported in Table 3 describe optimal agreement between potentiometric, spectrophotometric and NMR results. Even though the log *β* values obtained from NMR data are slightly different from the values obtained by potentiometric and spectrophotometric measurements (ca. 0.5 log units), they agree within the uncertainty of the calculation. This result is expected due to the reduced number of experimental data that can reasonably be collected by NMR titrations.

As well as it happens for the ligands, even when studying equilibria involving metal cations, their acid–base properties (i.e., their hydrolysis) must be considered in the speciation model, since many hydrolytic species, including polynuclear and mixed ones [52,53] may be formed, significantly affecting all other equilibria. In the case of Eu^3+^ (and other Ln^3+^ ions), the nature and stability of these species is still questionable. While the formation of the first [Ln(OH)]^2+^ species is nowadays well defined, and its hydrolysis constants may be easily found in literature in a wide range of conditions, few or no reliable data are available in relation to the nature and stability of other species. That is why Ekberg and Brown, who authored one of the most recent compilations of the hydrolysis constants of metal cations [54], suggest to include only the [Ln(OH)]^2+^ species in the speciation models for Ln^3+^, and for Eu^3+^ in particular. Concerning further hydrolytic Eu^3+^ species (mainly [Eu(OH)_2_]^+^ and [Eu(OH)_3_]), the same authors speculate about the (un)reliability of values reported in literature for their hydrolysis constants, but not about their existence. In agreement with Ekberg and Brown, though we also believe that the formation of further [Eu_p_(OH)_r_] species is highly probable, and due to the uncertainty about the nature and stability of other Eu^3+^ hydrolytic species than [Eu(OH)]^2+^, we followed these authors’ recommendation to consider only the latter species in the model. As such, in this work log we used *β_10-1_* = −8.02, calculated by Klungness and Byrne [55], who accurately investigated the first hydrolysis of several Ln^3+^ cations within wide ionic strength and temperature ranges.

#### 2.2.2. Formation and Stability of Eu(III)-CFA Species

**Figure 5:**
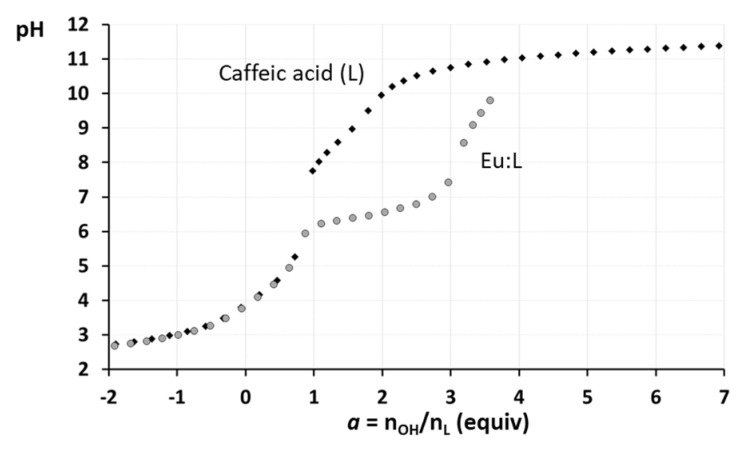
Representative potentiometric titration curves of CFA in the absence and presence of Eu^3+^ ions at 1:1 metal-to-ligand ratio (negative abscissae refers to the titration of strong acid added to the solutions to lower starting pH).

For the study of the Eu^3+^/CFA system, several independent potentiometric and UV-Vis spectrophotometric titrations were performed, at different Eu:CFA ratios in favour of the ligand, in order to try to enhance the possible formation of Eu*_p_*L*_q_*H*_r_*^(3*p*−3*q*+*r*)^ species with *q* > 1. The analysis of the obtained data shows that only 1:1 species are formed in the solution under our experimental conditions. The analysis of the potentiometric curves (Figure 5) shows that Eu^3+^ coordination by CFA starts at pH > 6, only when the deprotonation of the first catecholate hydroxyl group starts. This is evident by the superimposition of the potentiometric curves of Eu^3+^/CFA systems onto those of the free ligand. This is partial evidence that the carboxylate group, the first to be deprotonated, is not significantly involved in the coordination of the metal cation. Upon increasing pH, two more protons are subsequently displaced from the catecholate moiety, resulting in EuL type complexes, in agreement with several reported chelation studies of CFA-type ligands [15,56,57]. Furthermore, it is not surprising that the coordination of the lanthanide cation by the catecholate unit is preferred, as a five membered chelate ring is formed; this is a well-known coordination arrangement with higher stability when compared to the four-membered chelate rings that would be formed upon coordination via the carboxylic group.

**Figure 6:**
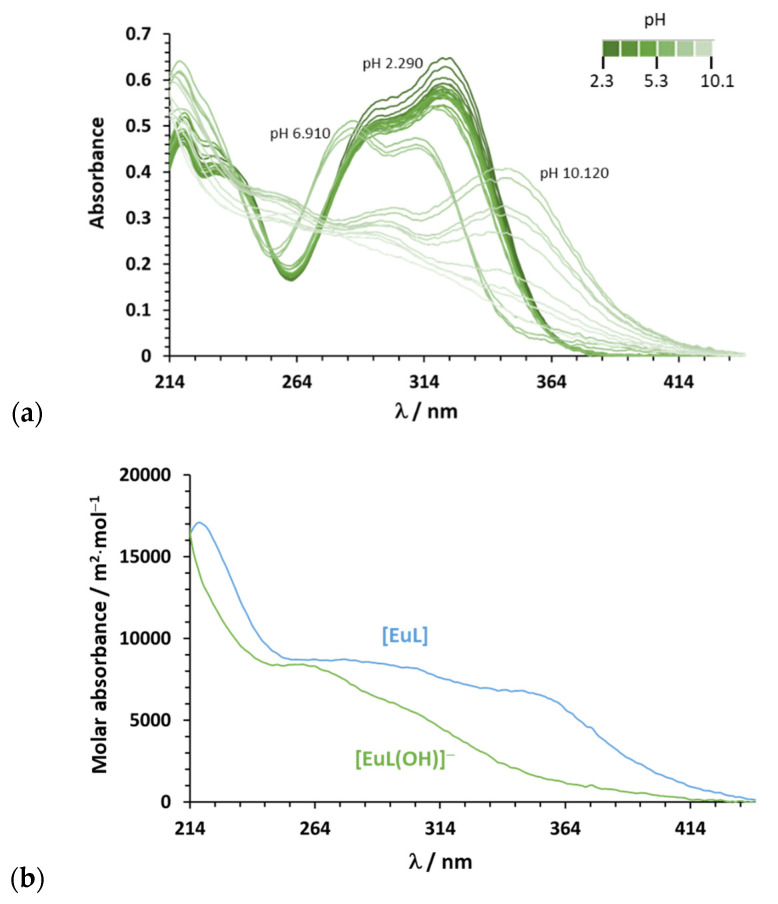
(**a**) Experimental absorption spectra of Eu^3+^/CFA system at 1:1 ratio, measured at different pH values, *c*_CFA_ = 4 × 10^−5^ mol·dm^−3^, in *I* = 0.2 mol·dm^−3^ KCl_(aq)_ and *T* = 298.15 K; (**b**) calculated molar absorbance spectra for the [EuL] and [EuL(OH)]^−^ species.

From the analysis of the potentiometric data, we could only determine the complex formation constant of [Eu(CFA)] species, with a log *β_110_* = 10.52 ± 0.02, due to the formation of sparingly soluble species at pH ≈ 7.0–7.5. This problem was partially solved by using UV-Vis spectrophotometric titrations, which gave us the possibility to work at lower concentrations, allowing measurements until pH ≈ 10.5–11, without evident precipitation. The use of UV-Vis spectroscopy also complemented the potentiometric measurements, allowing confirmation of the speciation model and double-checking of the formation constants determined. Several spectrophotometric titrations were performed at different M:L ratios (an example of one titration realized at a 1:1 ratio can be seen in Figure 6a). The analysis of UV-Vis spectra was not so straightforward, since there is no specific band resulting from the coordination with Eu^3+^. As such, we could only follow the changes on the ligand bands. However, a careful analysis of the UV-Vis spectra showed that an increase of pH leads to a bathochromic spectral shifting of the ligand peak. This was not surprising as it was also observed on the titrations of CFA alone, as a result of the deprotonation of the catecholate and carboxylic groups (see Figure S2). Nevertheless, in the presence of Eu^3+^ cation, the observed shift is followed by a decrease in the intensity of the spectra, which can be attributed to the coordination of the chromophore catechol moiety. Using HypSpec2014 software [50] we could process the obtained data, confirming the formation of the neutral [EuL] species. The possibility to investigate a wider (higher) pH range than that possible by potentiometry allowed further determination of the formation of the [EuL(OH)]^−^ hydroxo-species, resulting from the deprotonation of one water molecule from the [EuL] complex. The stability constants of [EuL] and [EuL(OH)]^−^ species are presented in Table 5, the former showing a perfect agreement between values obtained by both potentiometry and UV-Vis spectrophotometry. The molar absorbance spectra of these species were also calculated, and they are depicted in Figure 6b.

**Figure 7:**
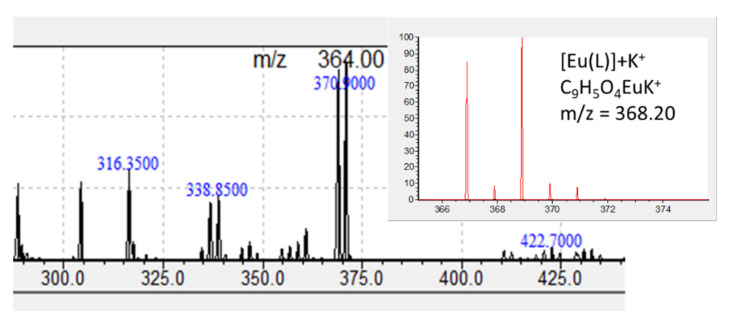
Experimental and theoretical (inset) ESI-MS spectra of the [Eu(L)]+K^+^ species in the Eu^3+^/CFA system in aqueous solution.

The formation of the [EuL] and [Eu(L)OH]^−^ species was also confirmed by ESI-MS measurements. Several samples were analysed, using different M:L ratios, in both positive and negative polarity modes. On negative mode spectra, a peak at *m/z* = 178.95 (Figure S4a) was observed, representative of the mono charged species LH_2_^−^ (C_9_H_7_O_4_^−^, *m/z* = 179.16), but also a peak at *m/z* = 347.00 (Figure S4b), which can be assigned to the [EuL(OH)]^−^ (C_9_H_6_O_5_Eu^−^, *m/z* = 346.11) species. The presence of the [EuL] species in the system was confirmed by a signal in the positive mode at *m/z* = 370.90 (Figure 7), corresponding to the K^+^ adduct of the mentioned species ([Eu(L)]+K^+^, C_9_H_5_O_4_EuK^+^, *m/z* = 368.20).

The mixed hydroxo-species [EuL(OH)]^−^ is very likely to be formed, particularly in relation to the fact that, as discussed above, Eu^3+^ may form further hydrolytic species in the pH of existence of [EuL(OH)]^−^ complexes. For the sake of correctness, it must be stated that the above-discussed exclusion from the model of other Eu*_p_*(OH)*_r_* species than [Eu(OH)]^2+^ (especially the possible [Eu(OH)_2_]^+^ and/or [Eu(OH)_3_] species) could lead to a slight overestimation of the stability constant of [EuL(OH)]^−^ species. Nevertheless, it must be taken into account that, as it is, the proposed speciation model perfectly interprets the experimental observations, giving consistent results both by potentiometric and spectrophotometric investigations. Therefore, the proposed speciation model for the Eu^3+^/CFA has to be regarded as reliable as soon as the hydrolytic scheme of Eu^3+^ is consistent; on the other hand, although this does not represent unequivocable proof of non-existence, further deprotonated [EuL(OH)*_r_*] species, with *r* > 1, were not even observed during ESI-MS experiments in solutions with a very high pH. However, this discussion highlights the absolute need for further investigations on the hydrolysis of Eu^3+^ and other Ln^3+^ cations, especially considering recent large numbers of speciation studies of aqueous systems involving them. These studies are ongoing, and will be the subject of future works.

Finally, it is also worth noting that the possibility of the formation of species in which the coordination of Eu^3+^ metal cations takes place by both the carboxyl and catechol sites—leading to the formation of oligomeric structures—was also taken into account during data analysis, as it is a common observation of the binding of caffeic acid and some derivatives of various divalent metal ions [17,56,58]. Nevertheless, the formation of this type of species was neither observed by potentiometry and spectrophotometry, nor by ESI-MS.

#### 2.2.3. Chemical Speciation and Sequestering Ability

**Figure 8:**
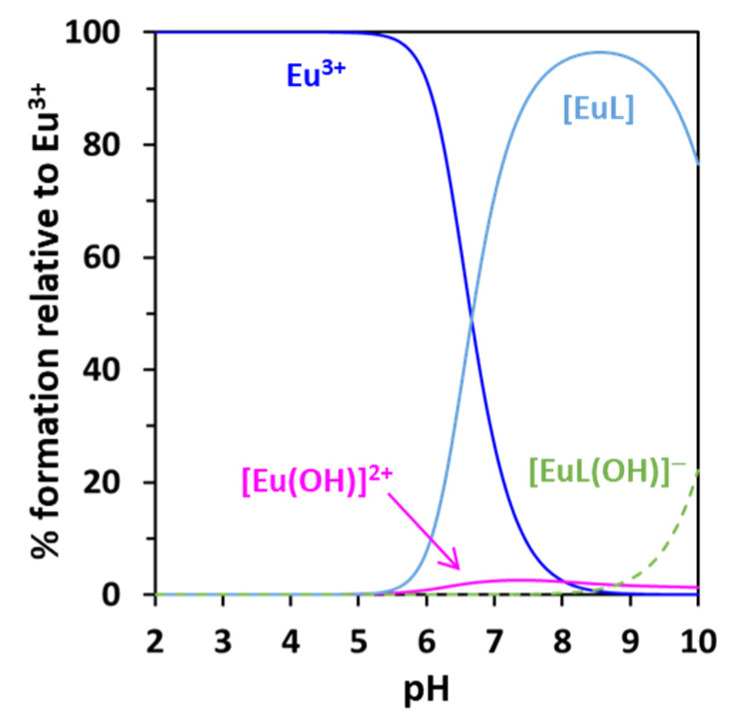
Distribution diagram of the Eu_p_L_q_H_r_ species as a function of pH in the Eu^3+^/CFA system (*c*_CFA_ = *c*_Eu3+_ = 1 mmol·dm^−3^), drawn using the determined UV-Vis set of constants. Dash line is used for the species detected only on UV-Vis spectrophotometric measurements.

Considering the chemical speciation of the CFA-Eu^3+^ system, we can see from Figure 8 that the coordination starts at pH > 6 with the formation of the [EuL] species; this remains the dominant species until pH ≈ 9, where the formation of the [EuL(OH)]^−^ becomes significant (represented using a dashed line, as it was only detected on UV-Vis spectrophotometric measurements).

From the distribution diagram presented, it is possible to state that CFA is a strong chelator for Eu^3+^, between pH ≈ 7 and 10, since almost all Eu^3+^ is complexed. Nevertheless, a meaningful evaluation can be only achieved if we compare the sequestering/chelating ability of CFA towards other trivalent metal cations, or the ability of similar ligands towards Eu^3+^. To the best of our knowledge, the only reliable data published considering the chelation of CFA with trivalent cations are in the work of Adams et al. [59], which reports a study of the solution equilibria between Al^3+^ and several 1,2-dihydroxyaryl ligands, including CFA, in aqueous solution, at *I* = 0.1 mol·dm^−3^ KCl_(aq)_ and *T* = 298.15 K.

There is also a more recent study from Beneduci et al. [60], but performed at *T* = 310.15 K. In contrast, due to the fast reduction of Fe^3+^ in aqueous solution in the presence of CFA, no data regarding Fe^3+^/CFA system were reported [61]. Concerning other ligands, it is worth comparing the chelation properties of CFA towards Eu^3+^ with those of the simple catechol system [62] and gallic acid (3,4,5-trihydroxybenzoic acid) [63] towards the same cation.

However, to compare different metal–ligand systems, the simple evaluation of the stability constants and/or the formation percentages of different metal–ligand complexes can be misleading, as we may have a different number and a different nature of complexes formed. In the different systems under comparison, we may have ligands with different denticities, different solution conditions, different acid–base properties, but also different metal cations. We may even have competition reactions between other metals and ligands simultaneously present, as protonation reactions and metal hydrolysis compete with the metal complex formation reactions [35,64,65,66,67].

**Figure 9:**
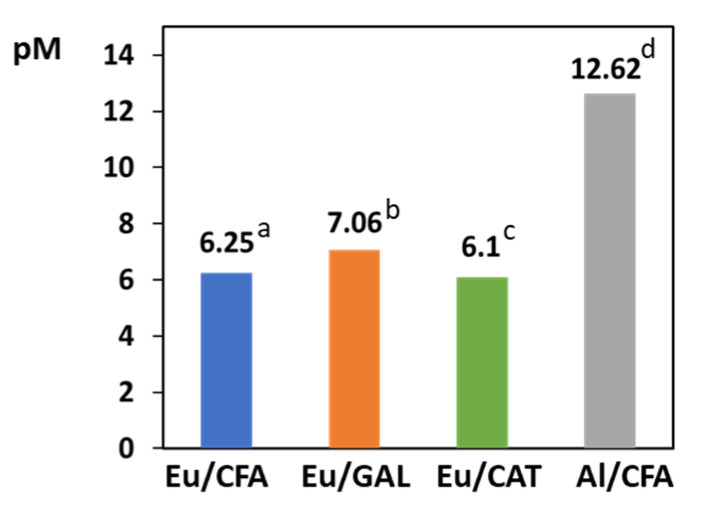
pM values calculated at pH = 7.4, *c*_M_ = 1 μmol·dm^−3^ and *c*_L_ = 10 μmol·dm^−3^, for several M/polyphenolic systems (M = Eu^3+^, Al^3+^), using data (a) determined in this work; reported in (b) [63]; (c) [62]; (d) [59].

To overcome these questions, several parameters have been proposed. One of these parameters is the pM value, commonly used in biological and medicinal chemistry to compare the affinity of different ligands for a specific metal under physiological conditions. This parameter is well-known and very often used for the comparison of the sequestering ability of different siderophores towards Fe^3+^ (being indicated as pFe in that particular case). pFe was defined by Raymond et al. in 1979 as −log[Fe^3+^], [Fe^3+^] being the concentration of the free aqueous ion [Fe(H_2_O)_6_]^3+^ present in the solution at specific experimental conditions (pH = 7.4, *c*_Fe_ = 1 μmol·dm^−3^ and *c*_L_ = 10 μmol·dm^−3^) [68]. As such, the higher the pM, the lower the concentration of free metal cation, and so, the better the chelation ability. In this work, based on the stability constants determined for our Eu^3+^/CFA system, we were able to determine the pEu parameter. This can be compared with the pM calculated for the other mentioned systems, using the stability constants reported (in particular, Eu^3+^/catechol (CAT) [62], Eu^3+^/gallic acid (GAL) [63], and Al^3+^/CFA [59]). The results are depicted in Figure 9.

The obtained pM values revealed a very similar chelating ability of the catecholate-type ligands towards Eu^3+^, with a slightly higher value for gallic acid. This result was expected considering that the coordination involved in all systems is very similar: the Eu^3+^ metal cation is coordinated by two adjacent hydroxyl groups from the aromatic ring (catecholate moiety). Considering the pAl calculated for the Al^3+^/CFA system, the absolute value is about double, which could appear as the result of better chelation ability of CFA towards Al^3+^. However, this result is just proof that pM value has to be used carefully. As already mentioned, pM value is determined by considering the concentration of the free metal cation in the solution, in specific conditions (pH = 7.4, *c*_M_ = 1 μmol·dm^−3^ and *c*_L_ = 10 μmol·dm^−3^). In this sense, this parameter is particularly robust when used to compare the systems of very strong chelators towards the metals in the study (such as siderophores towards Fe^3+^, usually having log *β* > 20), as there are very few (if any) other reactions competing with the complex formation reaction. In that case, the metal that is “not free” is bound to the ligand (siderophore) and we can, for example, easily compare the sequestering ability of several siderophores towards Fe^3+^. Unfortunately, the strength and greatness of this parameter—mainly represented by the easy-to-understand concepts behind it and the easiness of its calculation, even by non-experts—resulted in a sort of boomerang effect due to frequent “deviations” from Raymond’s original intentions and to its misuse, generating confusion in literature when conducting comparisons. For example, several pM values can be found in the literature for many systems, calculated in pH conditions and/or metal and ligand concentrations and ratios that significantly differ from each other, and from Raymond’s original; further details may be found, for example, in references [65,66]). In particular, in systems where the metal cation–ligand coordination is not so strong, other reactions (such as the hydrolysis of the metal cation) compete with the metal sequestration process, resulting in the formation of hydroxo-species. This leads to a still low concentration of the free metal cation in the system (resulting in a high pM value), but to a non-realistic evaluation of the sequestering ability of the ligand itself, as most or part of the metal is not bound to it [65]. This is exactly what we observe in the Al^3+^/CFA system: in the conditions of the pM calculation (pH = 7.4, *c*_Al_ = 1 μmol·dm^−3^ and *c*_L_ = 10 μmol·dm^−3^), the concentration of free Al^3+^ is very low (≈10^−13^ mol·dm^−3^), which results in a high pM value; however, only 52% of Al^3+^ is bound to CFA, with 48% of Al^3+^ being present in the form of Al(OH)*_x_* hydroxo-species.

To overcome this situation, a new parameter was defined by Sammartano and co-workers [67], called pL_0.5_. While pM is dependent on the concentration of the free metal, pL_0.5_ is determined on the basis of the concentration of the metal that is effectively bound to the ligand. In this way, it can be used for the comparison of systems with different speciation schemes; different ligands toward the same metal cation; the same ligand toward different cations; the same ligand/cation system under different conditions of pH, temperature, and ionic strength; and in the presence of other cations and ligands undergoing many different competing equilibria [67]. pL_0.5_ is then an empirical parameter that represents the ligand concentration necessary to sequester 50% of the metal cation when the latter is present in trace amounts (see Equation (3)).

**Figure 10:**
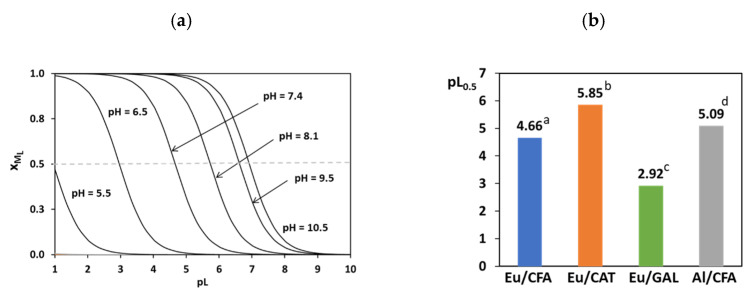
(**a**) Sequestration diagrams of Eu^3+^ by CFA at different pH values, at *I* = 0.2 mol·dm^−3^ in KCl_(aq)_, at *T* = 298.15 K. (**b**) pL_0.5_ values calculated at pH = 7.4 for several M/polyphenolic systems (M = Eu^3+^, Al^3+^) using data (a) determined in this work; reported in (b) [62]; (c) [63]; (d) [59].

The pL_0.5_ was then determined for the Eu^3+^/CFA system, at different pH values, and the individual sequestration diagrams are represented in Figure 10a, while the corresponding pL_0.5_ values for the chosen comparative systems at pH = 7.4 are shown in Figure 10b. From the diagrams in Figure 10a we can observe that the sequestering ability of CFA towards Eu^3+^ increases with the rise of pH, this growth being less remarkable at higher pH values. When compared with the same systems mentioned before, the behaviour between the different systems is very similar at pH = 7.4. In terms of absolute values, gallic acid (GAL) generally presents a higher sequestering ability for Eu^3+^ than the other two catecholate ligands (CFA and CAT), and CFA seems to be a good chelator for both Eu^3+^ and Al^3+^ at pH = 7.4.

### 2.3. Biological Studies

#### 2.3.1. Antimicrobial Activity

The minimum inhibitory concentration (MIC) values for the studied compounds and reference substances (EuCl_3_, gentamicin and fluconazole) are presented in Table 6. In general, Eu(III)-caffeinate showed higher antibacterial activity against *Escherichia coli* (Gram(−) bacteria) and *Bacillus subtilis* (Gram(+) bacteria) when compared with CFA and EuCl_3_ alone. This result is in agreement with the recently published review by Cota et al. [27], which reported that upon complexation with Ln^3+^ ions, the activity of the parent L is enhanced due to the increased lipophilicity, which allows Ln^3+^ complexes to penetrate through the lipid membranes of the cell and to cause a disturbance of the normal functioning of the cell, finally leading to cell death. This is usually explained by the Tweedy’s chelation theory and Overtone’s concept, which claims that cell permeability is highly dependent on the lipophilic character of their membrane, which favours the passage of only lipid soluble materials [27,69,70]. In our particular system we can assume an increase in lipophilicity after complexation, explained by the fact that the positive charge of the metal ion is partially shared with donor atoms, leading to a π-electron delocalization over the chelate ring [32,71].

Notwithstanding, the interaction of metal and/or ligand functional groups with cellular components via different paths can also change the cell wall properties, resulting in a higher toxicity of metal complexes as compared to a free ligand [72].

We can also observe that CFA and Eu(III)-caffeinate seem to be less active against the studied fungus (*Candida albicans*) when compared with their action on Gram(−) and Gram(+) bacteria. This was already observed for other Ln^3+^ complexes and it is usually explained by the different composition of the fungus cell wall, which makes it more difficult to penetrate [27]. Interestingly, in the case of alkali metal-caffeinates (e.g., potassium caffeinate), we observe a slightly higher activity for Gram(+) and a much lower activity for Gram(−) bacteria and fungi [10].

#### 2.3.2. Microelectrophoretic Mobility Measurements

**Figure 11:**
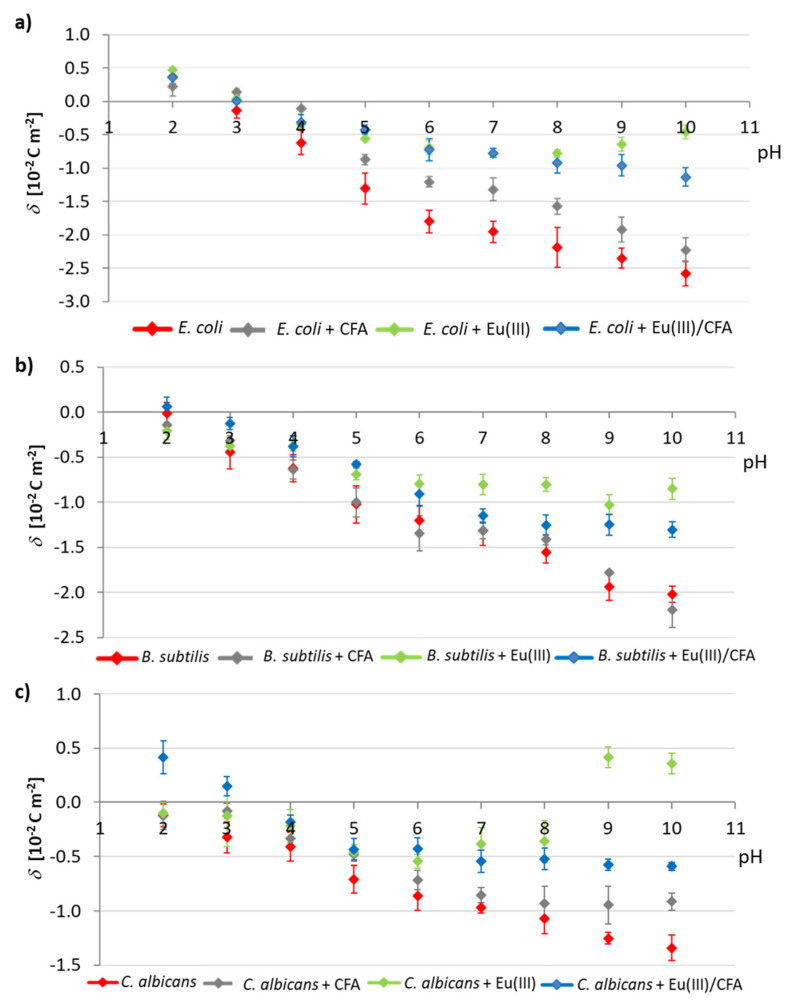
Influence of CFA, Eu(III) and Eu(III)/CFA system on the surface charge of: (**a**) *E. coli*, (**b**) *B. subtilis* and (**c**) *C. albicans* membranes.

As the interaction with the cellular membrane seems to be the main factor that explains the biological activity of CFA and its Eu(III) complexes, a detailed study was performed in order to obtain further insight into the compound–membrane interactions. This study was performed by electrophoretic mobility, a commonly used technique for surface characterization of nonbiological colloids and, more recently, bacterial, yeast or animal cells [73]. The determination of the surface charge of living cells (*δ*) is of prominent importance for understanding their behaviour and functions under various environmental conditions. The surface charge values of the *E. coli*, *B. subtilis* and *C. albicans* were measured as a function of pH in 0.2 mol·dm^−3^ KCl_(aq)_ solutions, containing only CFA or EuCl_3_, and EuCl_3_ with CFA at a 1:1 metal to ligand molar ratio (*c* = 10^−3^ mol·dm^−3^), as shown in Figure 11. Briefly, *E. coli* and *B. subtilis* were net negatively charged at pH values above their isoelectric points in CFA, Eu(III) and Eu(III)/CFA systems. Furthermore, it is obvious that at low pH values, no statistically significant changes were observed in the δ of membranes of any bacterium placed in the tested solutions compared with the control group (bacterium in the 0.2 mol·dm^−3^ KCl_(aq)_ only). The effect of the tested compounds on the δ values, due to their specific adsorption on the microbial membrane, becomes more significative as the pH increases. In our studies we observed a decrease in the negative surface charge, with the lowest values for: bacterium + CFA < bacterium + Eu(III) < bacterium + Eu(III)/CFA (see Figure 11). Interestingly, in the case of measurements performed with *C. albicans*, we obtained positive surface charge values for low pH in the solutions containing Eu(III)/CFA, and at high pH values for the solutions containing only Eu(III). The different membrane response to the presence of the metal complex can explain the lower activity observed against *C. albicans* when compared with the tested bacteria, in the MIC assays previously described.

Schott and Young [74] reported that polyvalent cations replace potassium and sodium ions originally bound to the carboxylate groups of the cell wall, and are firmly attached to these groups. Carboxylates coordinating di- and/or trivalent cations undergo little or no dissociation, as the formed bonds are stronger than in the case of K^+^ or Na^+^ coordination. This cation exchange process reduces the negative charge of the microbial membrane, which is consistent with our microelectrophoretic results. It is also reported that the metal binding on the microorganisms’ surface is probably the initial step in the uptake and concentration of the metals by the microbes, and in the toxic effects of these metals [75]. Our study showed significant pH-dependent changes in the surface charge values of microorganisms deposited on solutions containing Eu(III) and Eu(III)/CFA, suggesting a dependence on the interaction of the membrane surface with the species in a solution, i.e., on the chemical speciation. Considering the Eu(III)/CFA system, and in agreement with the speciation diagram presented in Figure 8, we can see that until pH ≈ 6, Eu(III) is free in a solution, and this explains the overlap of the measurements observed for the systems of microorganism/Eu(III) and microorganism/Eu(III)/CFA. At higher pH values, the coordination occurs, with the formation of a neutral [Eu(CFA)] species. Above pH 8–9, the formation of the negatively charged [Eu(CFA)(OH)]^−^ species changes the interaction with the tested membranes, as can be seen in Figure 11. The same is observed for the Eu(III) metal cation, where the effect is even higher, maybe due to a higher charge density. The toxicity of some metals to microorganisms varies with pH due to the hydrolysed species of these metals, which occur at higher pH values, bind to the cell surface and alter the net charge of the cell. Such a change in charge could affect various physiological functions of the cell, as well as its interactions with other cells and constituents present in the environment [75]. When analysing the three systems together, we can point out some relevant differences observed in the *C. albicans* membranes, which can explain the lower toxicity observed on MIC assays. Moreover, the obtained results can be also an indication that the speciation form of the metal cation, which depends on the presence of, for example, CFA and pH, rather than the type of microorganism, is a more relevant factor in determining changes in charge and membrane interactions, and hence, adsorption and toxicity.

#### 2.3.3. Antioxidant Activity

**Figure 12:**
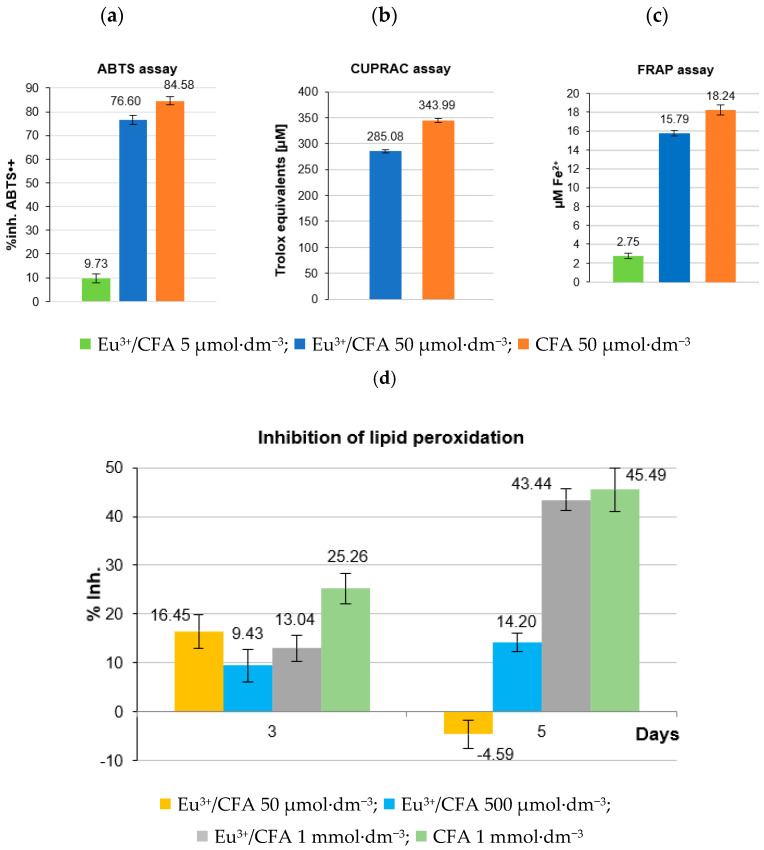
Antioxidant activity assays of CFA and Eu-CFA at indicated concentrations: (**a**) percentage of inhibition of ABTS^•+^ cation radicals; (**b**) results of CUPRAC assays expressed as Trolox equivalents [μmol·dm^−3^]; (**c**) evaluation of the reducing ability by FRAP assay; (**d**) percentage of inhibition of lipid peroxidation by Eu^3+^/CFA complex at the concentrations of 50, 500 and 1000 μmol dm^−3^. Mean values from three independent experiments ± SD are shown.

Besides the toxicity of the new metal complexes, we also studied how the high antioxidative potential of CFA was affected by the coordination with Eu(III). As such, we performed five independent antioxidant assays that showed that Eu(III) did not affect the antioxidant activity of CFA, or even decrease it (Figure 12). CFA exhibited strong antiradical activity against DPPH• (the obtained IC_50_ = 3.29 ± 0.29 μmol·dm^−3^) and ABTS•+ (% Inh. = 85%; *c* = 50 μmol·dm^−3^), whereas Eu(III)/CFA exhibited no activity toward DPPH• in the tested concentration; furthermore, the percentage inhibition of ABTS•+ was slightly lower than for acid alone (77%). For Eu(III)-caffeinate, it was not possible to determine the IC_50_ in DPPH assay because it precipitated at a higher concentration. Eu(III)/caffeinate complex showed reducing properties in CUPRAC and FRAP tests, but less than CFA (CUPRAC assay: 285.08 and 343.99 Trolox equivalents; FRAP assay: 15.79 and 18.24 μmol·dm^−3^ of Fe^2+^ for Eu(III)-caffeinate and the CFA complex and CFA, respectively). With the increase in the concentration of Eu(III)-caffeinate, its antioxidant activity increases as well. Studied compounds inhibited the peroxidation of lipids (in the test with linoleic acid). Until the third day, the differences between the compounds and their different concentrations were slight, but after the fifth day of measurement, Eu(III)-caffeinate at a higher concentration still possessed the antioxidant activity (% Inh. ≈ 43%), similarly to CFA (%Inh. ≈ 45%).

The antioxidant activity of phenolic acids is determined by the presence of the hydroxyl group (−OH) in the aromatic ring. The phenols with more than one −OH located in an ortho-position to each other, such as CFA, revealed the strongest antioxidant activity [76].

This is attributed to the lower energy of the O−H bond dissociation (BDE) that facilitates the donation of a hydrogen and/or an electron from the hydroxyl group to the reactive species (basicity). The antioxidant assays are generally based on electron (single electron transfer, SET) or hydrogen transfer (hydrogen atom transfer, HAT) or mixed mechanisms: sequential proton loss-electron transfer (SPLET) or single electron transfer-proton transfer (SET-PT) [77]. In DPPH• and ABTS•+ assays, antioxidants can react with radicals by a combination of SET and HAT mechanisms (i.e., the SPLET mechanism), whereas FRAP and CUPRAC assays are based on the SET mechanism [78]. Moreover, it was reported that SPLET is the most favourable mechanism of action in a polar solvent, while HAT dominates in a non-polar solvent (e.g., benzene) [79]. The pH of the medium may also affect the final results of the antioxidant assays. The CUPRAC test was conducted at pH ≈ 7 whereas the other assays were carried out at a lower pH. As is shown in our studies in a solution (Figure 8), at a pH < 6 the ligand remains almost uncoordinated, which causes the small difference in the results obtained for the complex and the ligand in FRAP, ABTS and lipid peroxidation assays.

## 3. Materials and Methods

### 3.1. Chemicals

Caffeic acid (3-(3,4-Dihydroxyphenyl)-2-propenoic acid, CFA), europium(III) chloride hexahydrate (EuCl_3_·6H_2_O), DPPH (2,2-diphenyl-1-picrylhydrazyl), ABTS (2,2-azino-bis(3-ethylbenzothiazoline-6-sulfonic acid), potassium persulfate (K_2_S_2_O_8_), TPZT (2,4,6-tris(2-pyridil)-s-triazine), iron(III) chloride hexahydrate (FeCl_3_∙6H_2_O), sodium acetate (C_2_H_3_NaO_2_∙3H_2_O), copper(II) chloride (CuCl_2_), ammonium acetate (CH_3_COONH_4_), neocuproine (2,9-dimethyl-1,10-phenanthroline), iron(II) sulfate (FeSO_4_), Trolox (6-hydroxy-2,5,7,8-tetramethylchroman-2-carboxylic acid), hydrogen peroxide (H_2_O_2_), gentamycin and fluconazole were purchased from Sigma-Aldrich Co. (St. Louis, MO, USA). Potassium hydroxide (KOH) and hydrochloric acid (HCl) TitraFix™ were purchased from POCH S.A. (Gliwice, Poland). KOH and HCl solutions were prepared by diluting the concentrated ampoules and were standardized against potassium hydrogen phthalate (Standard Reference Material, Merck, Germany) and tris (hydroxymethyl) aminomethane (Tris, ACS reagent, ≥99.8%, Sigma-Aldrich, St. Louis, MO, USA), respectively. Mueller-Hinton agar was supplied by Oxoid (Hampshire, UK). Methanol was sourced from Merck (Darmstadt, Germany). All chemicals had an analytical purity and were used without further purification. Ultrapure water was taken from a Milli-Q water (Millipore Direct-Q 3, Merck) purifying system and used in all experiments (R = 18 MΩ cm^−1^).

### 3.2. Synthesis and Characterization of Eu(III) Metal Complexes

A quantity of CFA in the amount of 0.003 mol was dissolved in a 0.1 mol⋅dm^−3^ aqueous solution of NaOH in a stoichiometric ratio of 1:1. The dissolution process was supported in an ultrasonic bath at *T* = 323.15 K. A clear solution of sodium caffeinate was obtained, to which an aqueous solution of EuCl_3_ with a concentration of 0.01 mol⋅dm^−3^ in a stoichiometric ratio of 3:1 (sodium caffeinate: europium chloride) was slowly added.

The resulting mixture was stirred at room temperature for 2 h and the mixture was then left for 48 h. The precipitated solid was filtered and washed with deionized water. The obtained complex was dried at *T* = 303.15 K for 3 days. For comparative reasons, the complexes of europium with cinnamic and *p*-coumaric acid were prepared following the same procedure.

Elemental composition of the prepared complexes was determined using a CHN 2400 PerkinElmer Analyzer. The following results were obtained: for europium caffeinate complex, Eu(C_9_H_7_O_4_)_3_·5H_2_O: C_exp_ = 40.30% (C_calc_ = 41.60%), H_exp_ = 3.65% (H_calc_ = 4.01%); for europium cinnamate, Eu(C_9_H_5_O_2_)_3_⋅6H_2_O: C_exp_ = 47.40% (C_calc_ = 47.04%), H_exp_ = 3.26% (H_calc_ = 3.07%); for europium *p*-coumarate, Eu(C_9_H_6_O_3_)_3_⋅4H_2_O: C_exp_ = 45.78% (C_calc_ = 45.97%), H_exp_ = 2.85% (H_calc_ = 3.00%). The europium caffeinate was also analysed using thermal analysis (TGA) using a Setsys 16/18 (Setaram). The resulting elemental formula of the complexes is: [Eu(CFA)_3_(H_2_O)_3_]·2H_2_O.

The structure of the metal complex of caffeic, cinnamic and *p*-coumaric acids, with europium, is described on the basis of spectroscopic studies, complemented with literature and X-ray data reported in the CSD crystallographic database. Infrared (IR) and Raman spectra (KBr and ATR techniques) were recorded with an Alfa spectrophotometer and a MultiRam, respectively, both from Bruker (Berlin, Germany).

### 3.3. Potentiometric Measurements

An Orion Star™ T900 Series Potentiometric Titrator (Thermo Scientific™, Waltham, MA, USA) equipped with an automatic burette and a combined glass electrodeOrion™ 8102BNUWP ROSS Ultra™ or Orion™ 8103BNUWP ROSS UltraTM (Thermo Scientific™) was used for potentiometric titrations (estimated accuracy ± 0.2 mV and 0.002 cm^3^ for the electromotive force reading and titrant additions, respectively). The potentiometric titrations were carried out using a double-walled glass cell connected to a water circulator as titration vessel. All titrations were conducted in an argon atmosphere, at the constant ionic strength of *I* = 0.2 mol⋅dm^−3^ KCl_(aq)_ and at *T* = 298.15 ± 0.1 K, in a pH range between ≈2 and 11 (or until observation of precipitation formation), using CO_2_-free KOH as a titrant.

The combined glass electrode was calibrated prior to each titration to determine the value of electrode potential (*E*^0^) and water dissociation constant (p*K*_w_). The calibration was carried out using a strong acid–strong base titration with no metal ion and ligand addition, in the presence of a background electrolyte in the same experimental conditions of ionic medium, strength and temperature (*I* = 0.2 mol⋅dm^−3^ in KCl_(aq)_, *T* = 298.15 ± 0.1 K). The total volume of the titration solution was 30.0 cm^3^ and the KOH solution was added in small increments to provide about 100 experimental data points for each run. The calibrations were performed in terms of free proton concentration (i.e., pH= −log [H^+^], not activity), as previously described [36,64,65]. For the investigation of the acid–base properties of the ligand, several solutions containing different concentrations of CFA (*c*_L_ ≈ 1.0 mmol⋅dm^−3^), known amounts of HCl to lower pH, and KCl were titrated with standardized KOH solutions. The same procedure was used for the study of metal–ligand interactions: several titrations were performed on solutions prepared as described above, plus suitable known amounts of Eu^3+^ to obtain different metal to ligand ratios (1:1 ≤ *c*_Eu_:*c*_L_ ≤ 1:3). Especially for experiments performed in the presence of metal cation, several trials were carried out to verify the reaching of equilibrium conditions, by checking and testing various equilibration times (up to several hours) and performing back-titrations.

### 3.4. UV-Vis Spectrophotometric Measurements

All UV-Vis spectrophotometric titrations were carried out using a Hitachi UV-Vis H3600U Spectrophotometer. Spectra were registered in the wavelength range of 210–500 nm. The measurements were performed in the same conditions as the potentiometric experiments (constant temperature of *T* = 298.15 ± 0.1 K, maintained by the use of a coupled water circulator, and at *I* = 0.2 mol⋅dm^−3^ in KCl_(aq)_). Spectrophotometric measurements were carried out directly in quartz cuvettes by titrating 2.5 cm^3^ of the titrant solution with standard KOH solution, up to pH ≈ 11. The pH of each solution was measured using a combined glass electrode Orion™ 8103BNUWP ROSS Ultra™, calibrated as previously described. For the evaluation of the formation of metal cation–ligand species and the determination of the corresponding stability constants, several measurements were carried out using CFA and EuCl_3_ solutions at different metal to ligand ratios (1:1 ≤ *c*_Eu_:*c*_L_ ≤ 1:3) and different concentrations (*c*_L_ ≈ 10^−5^ mol⋅dm^−3^).

### 3.5. H-NMR Measurements

^1^H-NMR experiments were performed using a Bruker Avance II 400 spectrometer. All the ^1^H-NMR experiments were carried out in solutions with a known ligand concentration of *c*_L_ ≈ 10^−3^ mol⋅dm^−3^, in KCl_(aq)_ at *I* = 0.2 mol⋅dm^−3^ and at *T* = 293.15 K, in 5 mm NMR tubes. A D_2_O capillary was introduced in each NMR tube as external reference. The use of a D_2_O capillary, instead of the preparation of the samples on a H_2_O/D_2_O mixture, avoids the need for evaluation of the isotopic effect, as the binding affinities of protonating groups are, in general, different for D^+^ and H^+^ [80]. Furthermore, a presaturation pulse sequence technique was used for water signal suppression. KOH standardized solution was used to adjust pH, measured using a calibrated combined Orion™ 8103BNUWP ROSS Ultra™ semi-micro tipped glass combination electrode.

### 3.6. ESI-MS Measurements

Electrospray-ionization mass spectrometry (ESI-MS) studies of Eu(III)/CFA system were carried out using a Shimadzu LC MS/MS 8040 spectrometer under the following conditions: mobile phase: 95% MeOH + 5% HCOOH (0.3% in H_2_O), flow rate: 0.3 cm^−3^⋅min^−1^, injection volume: 1 µdm^3^. Spectra were acquired in both positive and negative ion mode. The experiments were performed for solutions containing CFA alone and with EuCl_3_ at different metal to ligand molar ratios (1:1 ≤ *c*_Eu_:*c*_L_ ≤ 1:3), at *c*_L_ ≈ 10^−4^ mol⋅dm^−3^. Samples were freshly prepared and injected immediately after preparation.

### 3.7. Thermodynamic Calculations

Potentiometric titrations were processed using the non-linear least squares BSTAC4 computer program [81], which was used to determine the parameters (p*K*_w_, *E*^0^, acidic junction potential coefficient *j*_a_, analytical concentrations of reagents) of the electrode calibrations, the protonation constants of CFA, and the stability constants of its Eu(III) complexes. Spectrophotometric data were analysed by the HypSpec2014 program [50], which allowed the determination of the stability constants and the molar absorbance spectra of each absorbing species. From the same suite of programs, HypNMR was used to evaluate ^1^H-NMR data [51]. The HYSS [82] and ES4 [81] programs were used to draw the speciation and the sequestration diagrams.

The stability constants of Eu(III)/CFA complexes are expressed considering the overall equilibrium according to Equation (1):

*p* Eu^3+^ + *q* L^3−^ + *r* H^+^ = Eu*_p_*L*_q_*H*_r_*^(3*p*−3*q*+*r*)^       log *β_pqr_* (1)

The same equation is valid for the ligand protonation (*p* = 0) or Eu(III) hydrolysis constants (*q* = 0 and *r* < 0). Protonation constants are also expressed as stepwise equilibria according to Equation (2):

H^+^ + LH*_r_*_−1_^(*r*−4)^ = LH*_r_*^(*r*−3)^           log *K_0qr_* (2)

Formation constants, concentrations and ionic strengths are expressed in the molar concentration scale (*c*, mol⋅dm^−3^), and uncertainties are presented as ± standard deviation (SD). When not relevant, and for simplicity, the charges of the various species are omitted.

#### Evaluation of the Sequestering Ability

The evaluation of the sequestering ability of CFA (and other compared ligands) towards Eu^3+^ (and other cations) has been performed by the calculation of a semi-empirical parameter, pL_0.5_; this represents the total concentration of the ligand (L, as −log *c*_L_) necessary to bind 50% (as mole fraction, *x*_ML_ = 0.5) of a given component (M, in this case, Eu^3+^) in a given solution when M is present as trace (in our case, for calculation purposes, *c*_M_ = 10^−24^ mol⋅dm^−3^). Representing as *x*_ML_ the fraction of metal bound *only* to L vs. −log *c*_L_, we obtain a sigmoidal curve (sequestration diagram) that can be fitted to Equation (3), a Boltzmann-type equation, where pL = −log *c*_L_, and pL_0.5_ is the only adjustable parameter.

(3) $$x_{M_{L}}=\frac{1}{1+10^{\left(\mathrm{pL}-{\mathrm{pL}}_{0.5}\right)}}$$

From a quantification point of view, the higher the pL_0.5_, the greater the sequestering ability. Further details about this parameter, its genesis and meaning, the procedure to calculate it, and all the necessary equations are thoroughly described in reference [67]. Here, it is sufficient to mention that *x*_ML_ can be calculated by the mass-balance equation of the considered metal (M):

$$c_{M}=\left[M\right]+\underset{M_{L^{\prime }}}{\underbrace{\sum M_{p}{\left(\mathrm{OH}\right)}_{r}+\sum M_{p^{\prime }}{L^{\prime }}_{q^{\prime }}H_{r^{\prime }}}+}\;\underset{M_{L}}{\underbrace{\sum M_{p^{\prime \prime }}L_{q^{\prime \prime }}H_{r^{\prime \prime }}}+}$$

in which *c*_M_ is the total (analytical) concentration of the considered metal cation M, [M] is its free concentration, M_L_ represent the summation of the concentrations of all the species formed by the cation M with the desired ligand L, and M_L’_ stands for the concentration of all other species (including hydrolytic ones) formed by M with any other component and/or ligand different from L. Accordingly, *x*_ML_ is given by:

$$x_{M_{L}}=\frac{c_{M}-\left[M\right]-M_{L^{\prime }}}{c_{M}}$$

In the way it is calculated, pL_0.5_ represents the effective sequestering ability of the specific ligand L, “cleaned” of all competing reactions, and can thus be calculated in systems of all possible complexities, in which many other cations and ligands can be simultaneously present with those of interest (e.g., multicomponent real systems such as natural waters and biological fluids). Furthermore, with the sequestering ability being dependent on the conditions of the system under investigation (e.g., pH, ionic strength, temperature), so pL_0.5_ is. Of course, *x*_ML_ and, in turn, pL_0.5_ also depend on the analytical concentration of the metal (i.e., c_M_). However, as soon as c_M_ tends to 0 (i.e., at very low concentrations, such as those used in our calculations), pL_0.5_ usually tends to become a constant, and can be used for comparative purposes. In fact, despite Equation (3) not being fully rigorous and empirically derived from the sequestration diagrams, it proved excellent to model, in terms of sequestering ability, the behaviour of many systems of various complexity. Some other features of pL_0.5_, particularly in relation to similarities and differences with other approaches adopted for the same purposes, are discussed, for example, in reference [64,65,66,67].

### 3.8. Antimicrobial Studies

The MIC (Minimum Inhibitory Concentration) assay was determined by serial dilution of the test compound in an agar medium to which the appropriate inoculum of microorganisms was then added and incubated. The microorganisms used in the study came from the Polish Collection of Microorganisms (Wroclaw, Poland): *E. coli* (PCM 2857), *B. subtilis* (PCM 2850) and *C. albicans* (PCM 2566-FY). Microorganisms were grown overnight and then re-suspended in physiological solution to an optical density of 0.60 at λ = 600 nm (OD600), corresponding to 5.0 × 10^8^ CFU·cm^−3^ [50]. Microorganisms (0.1 cm^3^ of the reconstituted suspension) were seeded onto sterile Mueller-Hinton agar (containing 2% glucose) plates, to which previously suitable amounts of the tested compounds were added to give the desired concentration (0.01–35.0 mg·cm^−3^, solutions were prepared from the synthesized Eu(III)-CFA complex). Tested chemicals were dissolved in dimethyl sulfoxide (DMSO) solution. Negative controls were agar plates to which DMSO was added, and positive control were plates with gentamicin (in the case of bacteria) or fluconazole (in the case of fungi). The standards were tested in the range of concentrations from 0.25–6.00 mg.dm^−3^ in the case of gentamicin, and 0.10–1.09 mg.dm^−3^ for fluconazole. The plates were incubated at *T* = 347.15 K for 24 h. The lowest concentration without visible bacterial growth was determined as MIC.

### 3.9. Microelectrophoretic Mobility Measurements

*E. coli* (PCM 2857), *B. subtilis* (PCM 2850) and *C. albicans* (PCM 2566-FY) were harvested from the agar culture by centrifugation (14,000 rpm, 20 min) and washed five times with background solution (0.2 mol⋅dm^−3^ KCl_(aq)_) to remove any medium residues and to clean the microorganism suspensions before future analysis. The washed microorganisms were then suspended in the tested solutions to yield a final concentration of 2.5 × 10^7^ microorganisms⋅cm^−3^ and allowed to interact with the solution components for 1 h at room temperature. The tested solutions were 0.2 mol⋅dm^−3^ KCl_(aq)_ solutions containing CFA, EuCl_3_ or EuCl_3_ with CFA at 1:1 metal to ligand molar ratio, at *c* ≈ 10^−3^ mol⋅dm^−3^.

The electrophoretic mobility of the microbial cells was measured as a function of pH by a zeta potential analyser at 150 V (Zetasizer Nano ZS; Malvern Instruments Ltd., Malvern, UK) before being converted to surface charge density (*δ*) using the Equation (4), where *μ* is the electrophoretic mobility, *η* is the solution viscosity, and *d* is the diffuse layer thickness, previously determined using Equation (5) where *R* is the gas constant, *T* the experimental temperature, *F* is the Faraday constant, *I* is the ionic strength (0.2 mol⋅dm^−3^ in KCl_(aq)_), and *ε* and *ε*_0_ refer to the permittivity of free space and the relative permittivity of the medium [83].

(4) $$\delta =\frac{\eta \cdot \mu }{d}$$

(5) $$d=\sqrt{\frac{\varepsilon \cdot \varepsilon_{o}\cdot R\cdot T}{2\cdot F^{2}\cdot I}}$$

Both bacteria and fungi suspended in tested solution were titrated to the desired pH (range 2–10) using KOH at the same ionic concentration as the cell suspension. Six electrophoretic mobility measurements were made (each covering 100–200 runs with a duration of 2 s) for each pH value per sample. Each sample was measured in triplicate and values were presented as mean ± SD.

The sample cell used was the Malvern “Folded Capillary cell (DTS 1070)” of polyethylene, with 1 cm of optical length and coupled with electrodes. Aliquots of each cell suspension (1 cm^3^) were transferred to a Folded Capillary cell and the measurement was applied. Between each measurement, electrodes were rinsed with copious amounts of ethanol and Milli-Q water, followed by the tested microorganism suspension.

### 3.10. Anti-Oxidative Studies

All antioxidant assays were performed with samples prepared from the dissolution of the synthesized Eu(III)-CFA complex. All antioxidant assays were conducted in five repetitions for three independent experiments, and values were expressed as mean ± SD.

#### 3.10.1. DPPH Assay

The DPPH assay was conducted according to the Rice–Evans method described in reference [84]. Appropriate volumes of methanolic solutions of the tested compounds (*c* = 30 μmol·dm^−3^) were diluted in methanol in test tubes in order to prepare a series of solutions with various concentrations (final volume 1 cm^3^). Then, 2 cm^3^ of methanolic solution of DPPH• (*c* = 60 μmol·dm^−3^) was added to each tube. As a control, the same amount of DPPH• solution was added to a tube containing only methanol. After sample incubation for 60 min, at *T* = 296.15 K, in the dark, the absorbance at λ = 516 nm of the samples was measured on a Cary 5000 spectrophotometer (Varian, Santa Clara, CA, USA), against pure methanol. The ability to scavenge DPPH• radicals (% Inh) was calculated according to Equation (6):

% *Inh* = [(*A*_control_ − *A*_sample_)/*A*_control_] × 100% (6)

where: % Inh is the percentage inhibition of DPPH• radical, *A*_control_ is the absorbance of the control and *A*_sample_ represents the absorbance of the tested sample. The concentration was plotted against the percentage Inh and the IC_50_ value (concentration of compound (antioxidant) that inhibited 50% of DPPH• radical) was calculated from the obtained scavenging curves.

#### 3.10.2. ABTS Assay

The ABTS assay was performed as previously described [84]. The reagent was prepared by mixing aqueous solutions of ABTS (*c* = 7 mmol·dm^−3^) and K_2_S_2_O_8_ (*c* = 2.45 mmol·dm^−3^) in a volumetric ratio of 1:1, and left for 12–16 h at *T* = 296.15 K to generate ABTS•+ cation radicals. Then, 1 cm^3^ of the obtained ABTS•+ solution was mixed with 60 cm^3^ of methanol. An amount of 1 cm^3^ of ABTS•+ solution and 1 cm^3^ of methanolic solution of tested compounds (prepared at different concentrations, as described before) was mixed and incubated for 7 min at *T* = 296.15 K. The control contained methanol instead of the sample. The absorbance of the solutions was measured using UV-VIS spectrophotometer at λ = 734 nm against methanol (the reference). The ability to scavenge ABTS•+ cation radicals was expressed as the percentage Inh ABTS•+ and calculated according to the aforementioned Equation (6).

#### 3.10.3. FRAP Assay

The FRAP assay was carried out in accordance with the procedure described in the reference [85]. The FRAP reagent was prepared by mixing 250 cm^3^ of acetate buffer (*c* = 300 mmol·dm^−3^ at pH = 3.6), 25 cm^3^ of TPZT in 40 mmol·dm^−3^ HCl (*c* = 10 mmol·dm^−3^) and 25 cm^3^ of FeCl_3_ aqueous solution (*c* = 20 mmol·dm^−3^) (10:1:1 *v*/*v*/*v*). Then, 3 cm^3^ of the freshly prepared FRAP reagent was added to 0.4 cm^3^ of methanolic solution of tested compounds and mixed thoroughly. After 8 min of incubation at *T* = 296.15 K, the absorbance at λ = 595 nm was measured against a reference (containing 0.4 cm^3^ of methanol instead of the sample). Ferric reducing-antioxidant activity was expressed as Fe(II) ion equivalents (μmol·dm^−3^) using the calibration curve obtained over the range of 0.050–0.500 mmol·dm^−3^ of FeSO_4_ (calibration curve regression: y = (3.4 ± 0.2) x − 0.11 ± 0.02; *R^2^* = 0.9984).

#### 3.10.4. CUPRAC Assay

The CUPRAC assay was conducted according to reference [86]. An amount of 250 cm^3^ of CuCl_2_ (*c* = 10 mmol·dm^−3^ in water) was mixed with 250 cm^3^ of ammonium acetate (pH = 7 in water) and neocuproine (*c* = 75 mmol·dm^−3^ in methanol). Then, 3 cm^3^ of the CUPRAC mixture was added to 0.5 cm^3^ of methanolic solution of tested compound and 0.6 cm^3^ of distilled water. The samples were incubated for 60 min at room temperature (*T* = 296.15 K). The absorbance of the samples was measured at λ = 450 nm against a reference (containing 0.5 cm^3^ of methanol instead of the sample). Cupric reducing antioxidant activity was expressed as Trolox equivalents (μmol·dm^−3^) by using the calibration curve prepared over the range of 0.010–0.200 mmol·dm^−3^ of Trolox (y = (4575.8 ± 0.2) x + 0.027 ± 0.009; *R^2^* = 0.9919).

#### 3.10.5. Inhibition of the Lipid Peroxidation

The inhibition of the lipid peroxidation of the compounds under study was estimated by the use of the ferric thiocyanate method as described in reference [87]. The percentage inhibition of lipid peroxidation (% Inh) was also calculated according to the formula used during the DPPH assay (Equation (6)).

## 4. Conclusions

The europium sequestering ability of caffeic acid, an important natural derivative of abundant hydroxycinnamic acids, has been investigated in aqueous solutions and in solid state, in order to obtain some insights on the coordination mode of CFA towards Eu(III). It was observed that, while in aqueous solution, only 1:1 species are formed ([Eu(CFA)] and [Eu(CFA)(OH)]^−^), the solid formed within the synthetic procedure, was a 1:3 type of complex, with the proposed molecular formula of [Eu(CFA)_3_(H_2_O)_3_]·2H_2_O. Interestingly, in the Eu(III)-caffeinate complex, we observed a destabilization of the aromatic ligand system under the influence of metal. This effect is the opposite when compared with the coordination of Eu(III) by cinnamic or *p*-coumaric acid, where Eu(III) has a stabilizing effect on the aromatic system. This is probably due to the observed different mode of coordination of the metal cation that, in the case of CFA, occurs via catecholate moiety, while cinnamic and *p*-coumaric acid complex the metal via the carboxylate group.

Eu(III) complexation by CFA affects the biological properties of the ligand, including antioxidant and antimicrobial activity. Eu(III)/CFA complex showed higher antimicrobial activity toward selected Gram(+) and Gram(−) bacteria when compared to the ligand alone. Based on the findings of other authors [27], this can be explained by an increase in the lipophilicity of Eu(III) complex that may result in a higher interaction with the cell membrane, leading to the change in cell permeability and/or damage to the membrane integrity. This effect was observed to be pH dependent; this is in agreement with the sequestering ability of the ligand that increases with an increase in pH, resulting in the formation of a significant amount of Eu complexes. At higher pH values, the complexation of Eu(III) is more effective, so the changes of the membrane charge are more significant than in the case of free ligand. The lipophilicity does not seem to be an important parameter affecting the antioxidant properties of this complex in the conducted assays. The ability to donate a hydrogen or an electron to radical molecules is a crucial factor that determines whether a given chemical is a good or weak antioxidant. In the case of Eu(III)/CFA, the hydroxyl groups of the catechol moiety are engaged in metal–ion coordination, which diminishes the hydrogen/electron donating properties of the complex compared to the ligand, as was shown on the basis of chemical tests. These considerations are proved by the lower antioxidant activity of Eu(III)/CFA than CFA.

The experiments conducted in more realistic conditions (corresponding to living organisms), e.g., on cell lines, could give different conclusions about the antioxidant activity of studied compounds, due to the possibility of easier penetration of the complex through the cell membrane and the interaction with the cell oxidative system. Such studies will be undertaken in further research.

Finally, all of these conclusions could only have been achieved through multi-analytical-technique approaches, since single experiments could not give a comprehensive picture of the chemical and biological behaviour of the various investigated systems.

## Figures and Tables

**Table 1:** The wavenumbers (cm^−1^), intensities and assignments of bands observed in the experimental FT-IR (KBr and ATR) and FT-Raman spectra of CFA and Eu(III)-caffeinate.

CFA			Eu(III)-Caffeinate		Assignment	Varsanyi’s Designation [41]
IR_KBr_	IR_ATR_	Raman	IR_KBr_	IR_ATR_		
3431 s ^a^	3399 m		3421 vs	3396 m	ν(OH)_ar_^b^	
3235 m	3218 m		-	-	ν(OH)_ar_	
3000 w	3056 m	3032 vw	2963 w		ν(CH), ν(CH)_C=C_	20b
2839–2569 w					ν(OH)	
1647 vs	1634 s	1641 m	1630 s	1629 sh	ν(C=O), ν(CH)_C=C_	
1611 vs	1602 vs	1613 vs		-	ν(CC)	8a
1526 w	1526 m	1532 vw			ν(CC)	8b
			1501 m	1499 vs	ν_as_(COO)	
				1505 vs	ν_as_(COO)	
1450 vs	1442 vs	1453 vw			ν(CC)	19b
			1409 s	1409 s	ν_s_(COO)	
1364 w	1362 w	1353 w			ν(CC), β(OH)	14
		1299 s			β(OH)	
1283 vs	1271 s		1271 s	1271 vs	ν(C-OH)	
1215 s	1209 s				β(OH), β(CH)_C=C_	
1174 m	-	1186 w	1169 w	1170 m	β(CH)	18a
1115 m	1110 s	1108 vw	1119 m	1120 m	β(CH)	18b
			982 w	980 w	β_s_(COO)	
972 w	965 m	974 vw	961 w	977 w	γ(CH)_C=C,_ γ(CH)	17b
936 w	939 w			932 w	γ(CH)	17a
901 w	892 s				ν(CCO)	
856 w	853 m	852 vw	861 w	853 vw	γ(CH)	5
812 w					β(C=O)	
			816 w	823 w	α(CCC)	1
801 w	806 s	803 vw		793 m	γ(CH)	10a
787 w	788 m	779 vw		756 sh	α(CCC)	12
			701 w	695 m	γ_s_(COO)	
699 w	695 w	686 vw			γ(C=O)	
645 w	642 m			664 vw	φ(CC)	16a
603 w		603 vw	603 w	595 s	α(CCC)	6a
575 m		534 vw	535 w		γ(OH)	
459 w		459 vw	547 vw	547 s	α(CCC)	6b
409 w		446 vw	407 vw		φ(CC)	16b

^a^ s-strong; m-medium; w-weak; v-very; sh-shoulder. ^b^ the symbol “ν” denotes stretching vibrations. “β” denotes in-plane bending modes. “ γ” designates out-of-plane bending modes; “φ(CCC)” denotes the aromatic ring out-of-plane bending modes and “ α(CCC)” designates the aromatic ring in-plane bending modes.

**Table 2:** The wavenumbers (cm^−1^), intensities and assignments of bands observed in the experimental FT-IR (KBr and ATR) spectra of cinnamic acid, Eu(III)-cinnamate and *p*-coumaric acid, and Eu(III)-*p*-coumarate.

IR_KBr_	IR_ATR_	IR_KBr_	IR_ATR_	Assignment	Varsanyi’s Designation [41]
Cinnamic acid		Eu(III)-cinnamate			
1684 vs	1667 vs	-	-	ν(C=O)	
-	-	1536 s	1520 s	ν_as_(COO^−^)	
1494 w	1492 m	-	1507 s	β (CH)_aro._	19 a
1448 m	1449 m	1448 s	1446 m	ν(CC)_aro_+ β(CH)_aro._	19 b
-	-	1412 vs	1399 s	ν_s_(COO^−^)	
1073 w	1071 m	1069 w	1071 vw	ν(CC)_aro_+ β(CH)_aro._	18b
1025 w	1030 w	1029 w	1026 vw	ν(CC)_aro_+ β(CH)_aro._	18a
*p*-coumaric acid		Eu(III)-*p*-coumarate			
3422 vs	3330 w	3423 vs	-	ν(OH)_aro._	
2924 w- 2580 w	2944 w-2379 m	2924 w- 2613 vw	2804 m	ν(OH)_aro._	
1671 s	1664 vs	-	-	ν(C=O)	
-	-	1559 m	1559 m	ν_as_(COO^−^)	
1511 m	1503 m	1512 s	1502 vs	ν(CC)_aro_	19a
1448 m	1440 s	1439 m	1442 m	ν(CC)_aro_	19b
-	-	1409 s	-	ν_s_(COO^−^)	
1106 w	1105 m	1104 w	1107 m	β(CH)	18b
1063 w	-	-	-	β(CH)	18a

**Table 3:** Protonation constants ^a^ of CFA in KCl_(aq)_ at *I* = 0.2 mol·dm^−3^ and *T* = 298.15 K.

Species	(*p:q:r*)	log *β_pqr_* ^a^ (log *K_01r_*) ^b^
LH	0:1:1	11.93 ± 0.08
LH_2_	0:1:2	20.59 ± 0.08 (8.66) (20.47 ± 0.06) ^c^ (20.9 ± 0.3) ^d^
LH_3_	0:1:3	24.92 ± 0.01 (4.33) (24.97 ± 0.03) ^c^ (25.3 ± 0.3) ^d^

^a^ log *β_pqr_* refers to the equilibrium: *p*M + *q*L + *r*H^+^ = M*p*L*q*H*r*; ^b^ log *K_01r_* refers to the equilibrium: H^+^ + LH_*r*−1_ = LH*_r_*, ± SD; ^c^ values obtained from spectrophotometric measurements; ^d^ values obtained from ^1^H-NMR measurements.

**Table 4:** Calculated chemical shift (δ/ppm) of different (de)protonated species of CFA, in KCl_(aq)_ at *I* = 0.2 mol·dm^−3^ and *T* = 298.15 K.

Nucleus	δ/ppm			
	L	LH	LH_2_	LH_3_
H_b_	7.2419	7.2429	7.2612	7.6202
H_c_	6.9927	7.035	7.1463	7.1989
H_e_	6.8656	6.918	7.0582	7.1294
H_d_	6.6373	6.701	6.9168	6.9433
H_a_	6.1865	6.2177	6.3258	6.3534

**Table 5:** Eu(IIII)/CFA complex formation constants in KCl_(aq)_ at *I* = 0.2 mol·dm^−3^ and *T* = 298.15 K.

Species	(*p*:*q*:*r*)	log *β_pqr_* ^a^
EuL	1:1:0	10.52 ± 0.02 (10.57 ± 0.03) ^b^
[EuL(OH)]^−^	1:1:−1	-- (0.03 ± 0.05) ^b^

^a^ log *β_pqr_* refers to the equilibrium: *p*Eu + *q*L + *r*H^+^ = Eu*_p_*L*_q_*H*_r_* ± SD; *^b^* values obtained from the fitting of the spectrophotometric measurements.

**Table 6:** The minimum inhibitory concentration (MIC) values for tested compounds against selected microorganisms.

Microorganism	MIC			
	mg·cm^−3^ (mmol·dm^−3^)			
	CFA	Eu-Caffeinate	EuCl_3_	Positive Control
*Escherichia coli* Gram(−) bacteria	13 (72.2)	4 (5.1)	10 (27.3)	Gentamicin 0.0005 (0.001)
*Bacillus subtilis* Gram(+) bacteria	18 (99.9)	4 (5.1)	12 (32.8)	Gentamicin 0.005 (0.01)
*Candida albicans* (fungi)	29 (161.0)	>4 (>5.1)	6 (16.4)	Fluconazole 0.00025 (0.00082)

## Supplementary Materials

The following are available online at https://www.mdpi.com/article/10.3390/ijms23020888/s1.
