# Supplementary material for: Caffeic Acid/Eu(III) Complexes: Solution Equilibrium Studies, Structure Characterization and Biological Activity
Source: Int J Mol Sci. 2022 Jan 14;23(2):888. doi: 10.3390/ijms23020888 (PMC8775996; doi:10.3390/ijms23020888)
Supplement: Supplementary file 1 [file ijms-23-00888-s001.zip › ijms-1526888-supplementary.pdf]

Supplementary materials for

Caffeic acid / Eu(III) complexes: solution equilibrium studies, structure characterization and biological activity

Żaneta Arciszewska [1], Sofia Gama[1,*], Monika Kalinowska[2], Grzegorz Świderski[2], Renata Świsłocka [2], Ewelina Gołębiewska [2], Monika Naumowicz [3], Mateusz Worobiczuk [3], Adam Cudowski [4], Anna Pietryczuk [4], Concetta De Stefano [5], Demetrio Milea [5], Włodzimierz Lewandowski [6] and Beata Godlewska-Żyłkiewicz [1,*]

1 Department of Analytical and Inorganic Chemistry, Faculty of Chemistry, University of Bialystok, K. Ciołkowskiego 1K, 15–245 Białystok, Poland; z.arciszewska@uwb.edu.pl (Ż.A.); sofia.gama@uwb.edu.pl (S.G.); bgodlew@uwb.edu.pl (B.G.-Ż.);

2 Department of Chemistry, Biology and Biotechnology, Bialystok University of Technology, Wiejska 45E, 15–351 Białystok, Poland; m.kalinowska@pb.edu.pl (M.K.); g.swiderski@pb.edu.pl (G.Ś.); r.swislocka@pb.edu.pl (R.Ś.); e.golebiewska@pb.edu.pl (E.G);

3 Department of Physical Chemistry, Faculty of Chemistry, University of Bialystok, K. Ciołkowskiego 1K, 15–245 Białystok, Poland; monikan@uwb.edu.pl (M.N.); Mateusz Worobiczuk, mworobiczuk@gmail.com (M.W.);

4 Department of Water Ecology, Faculty of Biology, University of Bialystok, K. Ciolkowskiego 1J, 15-245 Białystok, Poland; cudad@uwb.edu.pl (A.C.); annapiet@uwb.edu.pl (A.P.);

5 Dipartimento di Scienze Chimiche, Biologiche, Farmaceutiche ed Ambientali, CHIBIOFARAM, Università degli Studi di Messina, V.le F. Stagno d'Alcontres, 31, 98166 Messina, Italy; cdestefano@unime.it (C.D.S.); dmilea@unime.it (D.M.);

6 Prof Waclaw Dabrowski Institute of Agricultural and Food Biotechnology - State Research Institute, 36 Rakowiecka St., 02-532 Warsaw, Poland; w.lewandowski@pb.edu.pl (W.L.);

* Correspondence: sofia.gama@uwb.edu.pl (S.G.), +48857388090; bgodlew@uwb.edu.pl (B.G.-Ż.), +48857388257

List of acronyms

ABTS	2,2-azino-bis(3-ethylbenzothiazoline-6-sulfonic acid
BDE	Bond Dissociation Energy
CAT	Catechol
CFA	Caffeic Acid
CUPRAC	Cupric Reducing Antioxidant Power
DMSO	Dimethyl sulfoxide
DNA	Deoxyribonucleic Acid
DPPH	2,2-diphenyl-1-picrylhydrazyl
EA	Elemental Analysis
ESI-MS	Electrospray Ionization Mass Spectrometry
FRAP	Ferric Reducing Antioxidant Power
FT-IR	Fourier-Transform Infrared Spectroscopy
FT-Raman	Fourier-Transform Raman spectroscopy
GAL	Gallic Acid (3,4,5-trihydroxybenzoic acid)
HAT	Hydrogen Atom Transfer
IR	Infrared
MIC	Minimum Inhibitory Concentration
NMR	Nuclear Magnetic Resonance
SD	Standard Deviation
SET	Single Electron Transfer
SET-PT	Single Electron Transfer-Proton Transfer
SPLET	Sequential Proton Loss-Electron Transfer
TGA	Thermal Gravimetric Analysis
UV-Vis	Ultraviolet-Visible

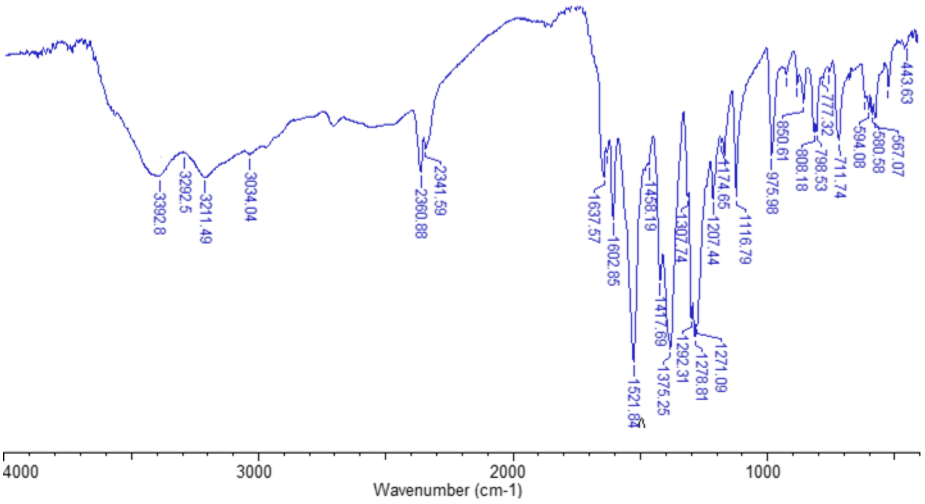

Figure S1. FT-IR (IR_{KBr}) spectra of CFA sodium salt.

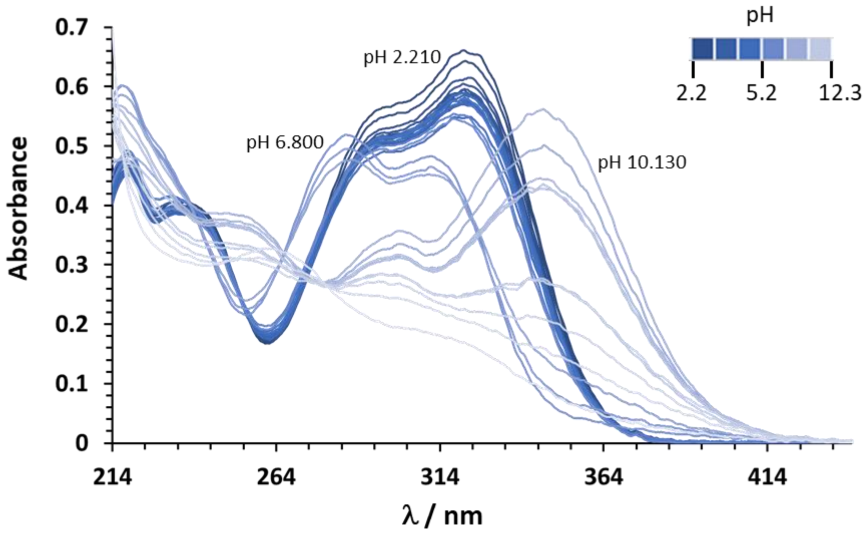

a)

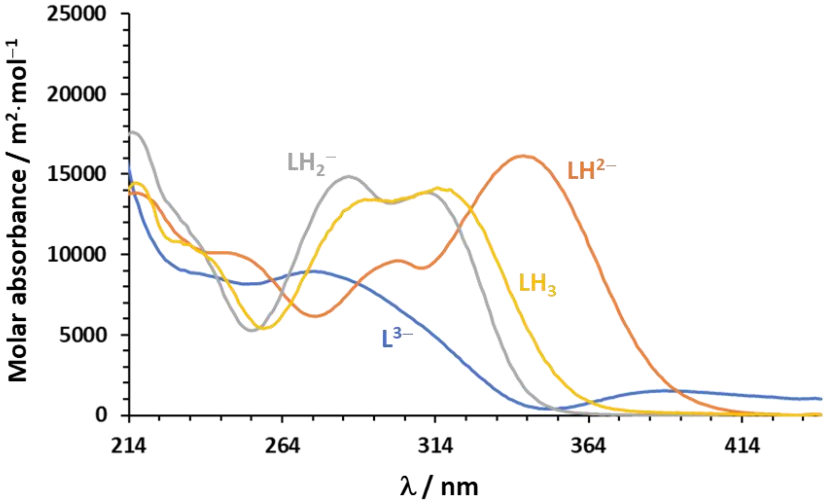

b)

Figure S2. a) Representative experimental UV-Vis spectrophotometric titration of CFA and b) calculated molar absorbance spectra for the individual protonated CFA species [15].

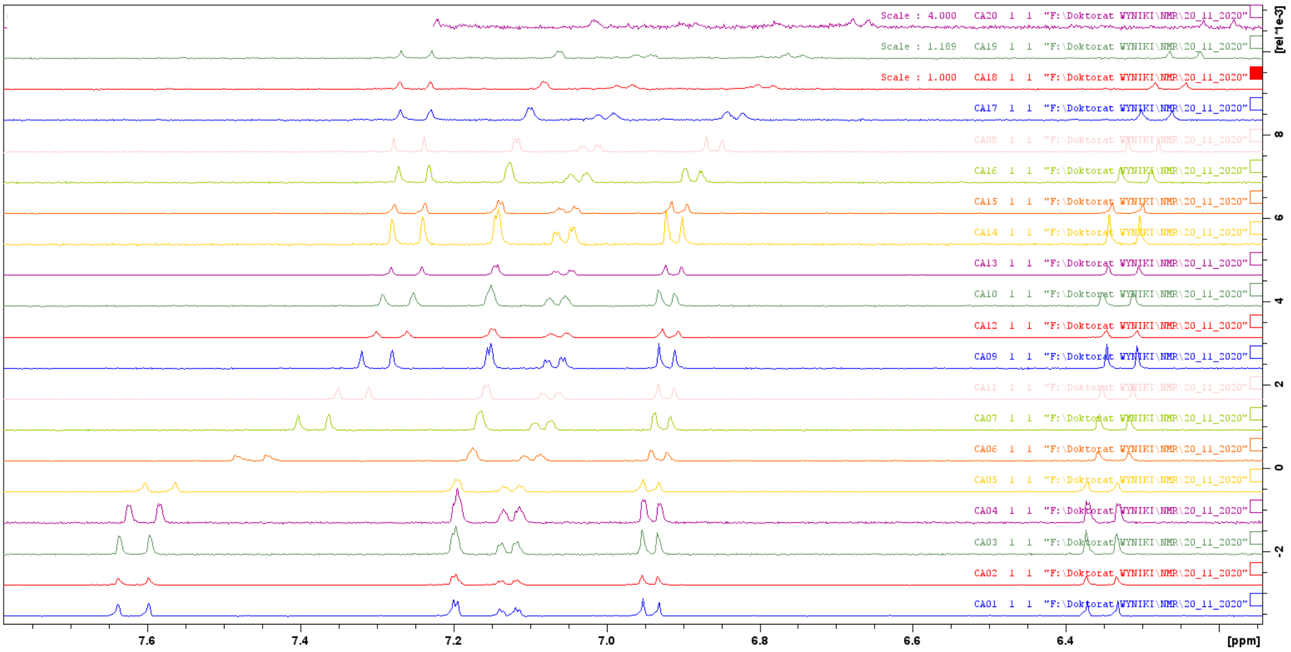

Figure S3. ^{1}H-NMR spectra of the free ligand obtained at different pH values, from pH =2.33 to pH =11.00. c_{CFA} = 1 mmol·dm^{-3}

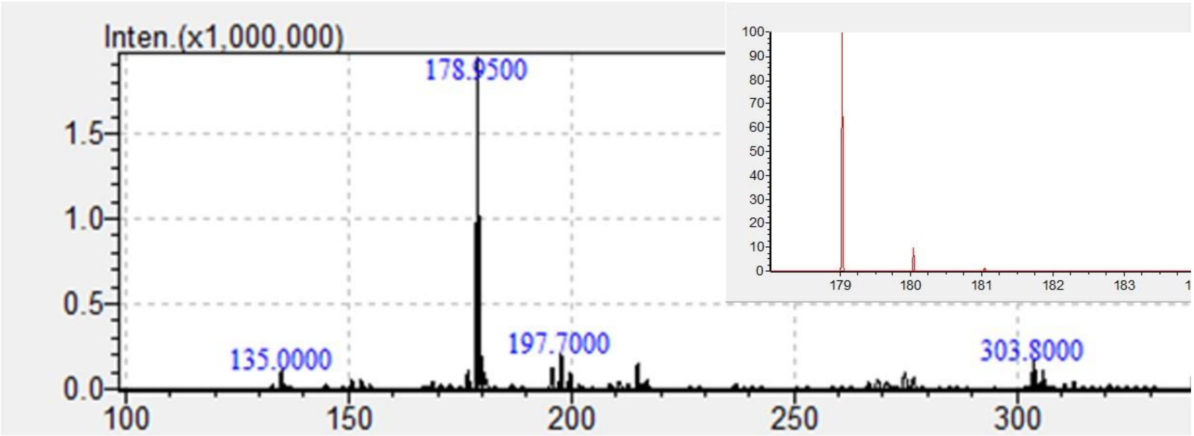

a) LH_2^-, $C_9H_7O_4^-$, m/z = 179.16

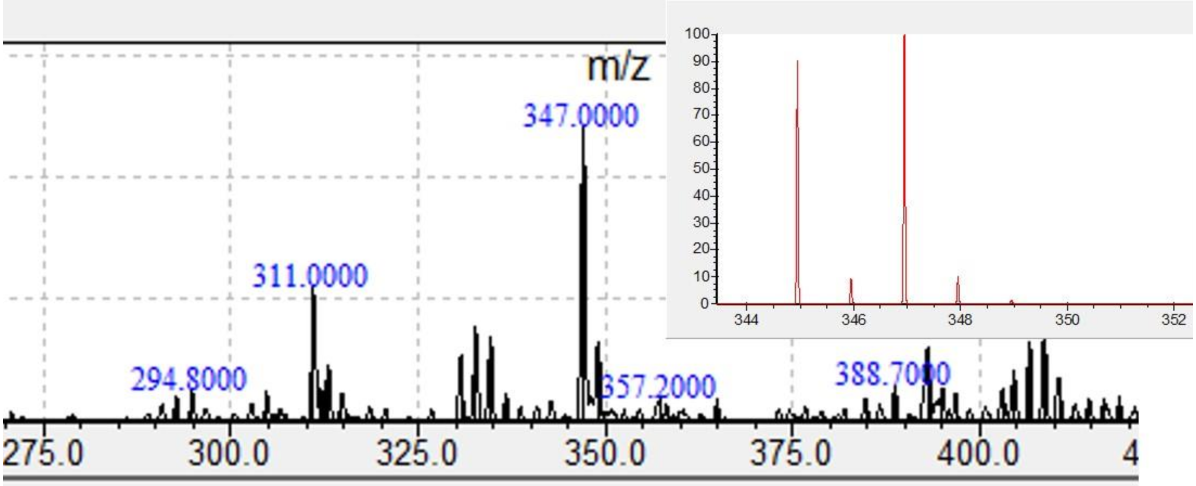

b) $[EuL(OH)]^-$, $C_9H_6O_5Eu^-$, m/z = 346.11

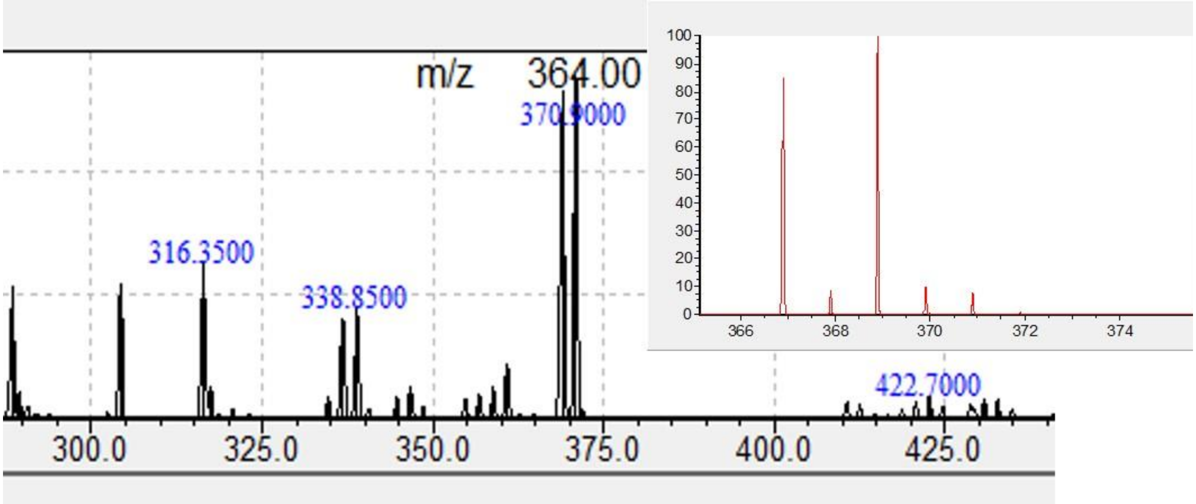

c) $[Eu(L)]+K^+$, $C_9H_5O_4EuK^+$, m/z = 368.20

Figure S4. Experimental and theoretical (inset) ESI-MS spectra of the a) LH_2^- , b) $[EuL(OH)]^-$ and c) $[Eu(L)]+K^+$ species in the Eu^{3+}/CFA system in aqueous solution.

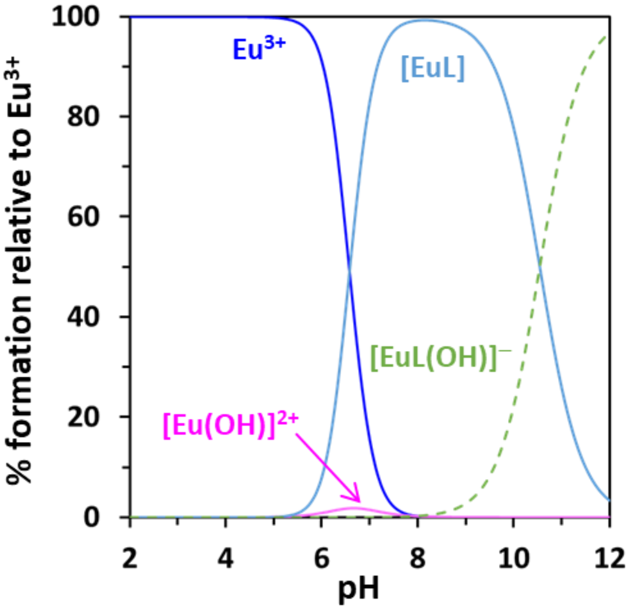

Figure S5. Distribution diagram of $Eu_pL_qH_r$ species as a function of pH in the Eu^{3+}/CFA system ($c_{CFA} = 2 \times c_{Eu3+} = 1$ mmol.dm^{-3})

Table S1 Experimental chemical shift (δ / ppm) of the indicated nucleus at different pH values. c_{CFA} = 1 mmol·dm^{-3}

pH	δ / ppm				
	**Ha**	**Hb**	**Hc**	**Hd**	**He**
2.34	6.353	7.621	7.202	6.944	7.131
2.47	6.352	7.617	7.199	6.944	7.130
2.68	6.354	7.617	7.197	6.943	7.129
3.14	6.353	7.607	7.196	6.943	7.126
3.52	6.355	7.586	7.200	6.944	7.125
4.23	6.341	7.464	7.175	6.933	7.099
4.81	6.338	7.385	7.165	6.926	7.084
5.12	6.333	7.332	7.157	6.923	7.074
5.26	6.327	7.301	7.154	6.921	7.066
5.7	6.328	7.282	7.150	6.918	7.063
5.92	6.334	7.275	7.153	6.922	7.065
6.6	6.324	7.263	7.145	6.913	7.056
6.73	6.322	7.261	7.142	6.912	7.054
7.38	6.319	7.260	7.142	6.907	7.051
8.00	6.308	7.255	7.129	6.889	7.039
8.54	6.298	7.259	7.117	6.859	7.022
8.73	6.281	7.252	7.099	6.824	6.997
9.14	6.263	7.254	7.083	6.793	6.979
9.38	6.246	7.247	7.063	6.755	6.951
9.63	6.235	7.245	7.050	6.736	6.937
10.39	6.214	7.246	7.029	6.692	6.912
10.8	6.206	7.243	7.019	6.676	6.899
11.02	6.202	7.241	7.018	6.671	6.893
11.03	6.203	7.245	7.018	6.668	6.896
11.46	6.192	7.238	7.003	6.657	6.882
12.09	6.192	7.244	6.994	6.643	6.868

Table S2. Calculated molar absorbance spectra for the individual species of CFA [15] and Eu^{3+}/CFA system.

λ / nm	L^{3-}	LH^{2-}	LH_2^-	LH_3	EuL	$[EuL(OH)]^-$
210	20621	13023	16052	12130	13636	22875
211	18876	13298	16569	12656	14589	20811
212	17588	13484	16906	13227	15347	19074
213	16371	13638	17252	13716	15922	17625
214	15354	13757	17491	14089	16405	16399
215	14500	13805	17610	14333	16718	15487
216	13727	13824	17566	14456	16947	14861
217	13186	13807	17466	14426	17090	14350
218	12554	13761	17248	14351	17104	13826
219	12056	13686	16948	14198	17008	13368
220	11572	13562	16488	13875	16907	13043
221	11155	13428	15930	13396	16722	12775
222	10820	13293	15369	12982	16467	12481
223	10494	13086	14766	12314	16139	12150
224	10188	12801	14176	11766	15853	11894
225	9933	12515	13726	11384	15542	11625
226	9687	12167	13359	11083	15191	11340
227	9576	11968	13193	10979	14833	11077
228	9404	11677	12928	10837	14527	10839
229	9289	11399	12704	10820	14186	10614
230	9150	11219	12447	10802	13799	10393
231	9055	11034	12125	10800	13454	10180
232	9059	10918	11961	10630	13084	9976
233	8985	10778	11756	10619	12697	9780
234	8902	10617	11366	10617	12326	9570
235	8888	10446	11144	10463	11986	9432
236	8867	10373	10904	10293	11708	9294
237	8847	10296	10668	10184	11403	9195
238	8804	10196	10348	10103	11068	9074
239	8762	10136	9974	9961	10754	8932
240	8706	10121	9540	9793	10520	8795
241	8675	10102	9198	9686	10293	8721
242	8629	10113	8854	9489	10050	8636
243	8590	10100	8485	9267	9846	8556
244	8526	10095	8056	8965	9700	8509
245	8455	10099	7515	8603	9554	8447
246	8395	10099	7121	8344	9379	8394
247	8353	10090	6762	8031	9243	8363
248	8313	10063	6474	7774	9174	8391
249	8265	10020	6109	7398	9079	8406
250	8234	9950	5774	6972	8948	8356
251	8197	9897	5578	6722	8853	8332
252	8195	9809	5401	6378	8806	8339
253	8188	9731	5302	6142	8786	8390
254	8206	9632	5262	5945	8738	8402
255	8198	9481	5283	5666	8715	8395
256	8213	9331	5360	5522	8729	8415

257	8231	9165	5475	5441	8722	8402
258	8276	9015	5645	5385	8721	8412
259	8336	8755	5930	5451	8712	8424
260	8408	8532	6257	5471	8713	8394
261	8439	8396	6466	5569	8698	8340
262	8498	8172	6807	5713	8713	8312
263	8553	7928	7210	5930	8737	8316
264	8592	7666	7663	6183	8739	8302
265	8689	7414	8185	6462	8706	8222
266	8727	7201	8622	6727	8686	8128
267	8773	7006	9074	6985	8679	8069
268	8814	6836	9496	7360	8694	8027
269	8854	6633	10041	7726	8688	8007
270	8907	6475	10577	8175	8659	7925
271	8924	6356	11012	8519	8673	7851
272	8954	6273	11433	8843	8700	7793
273	8961	6202	11827	9200	8712	7709
274	8977	6172	12333	9655	8720	7635
275	8970	6154	12762	9967	8744	7546
276	8941	6184	13115	10406	8745	7468
277	8938	6237	13457	10791	8715	7363
278	8905	6302	13739	11018	8678	7215
279	8844	6417	14019	11393	8678	7159
280	8814	6529	14236	11586	8683	7090
281	8750	6699	14428	11948	8646	6980
282	8694	6843	14563	12149	8603	6855
283	8632	7005	14660	12339	8568	6780
284	8578	7240	14768	12675	8587	6736
285	8514	7443	14827	12795	8591	6671
286	8442	7636	14848	12892	8567	6584
287	8358	7853	14797	13075	8542	6517
288	8284	8050	14741	13193	8533	6449
289	8196	8256	14653	13300	8496	6383
290	8114	8468	14517	13347	8485	6335
291	8011	8630	14338	13416	8476	6298
292	7908	8791	14136	13398	8459	6235
293	7792	8931	13937	13383	8420	6141
294	7680	9064	13740	13426	8383	6086
295	7555	9162	13565	13384	8380	6085
296	7448	9255	13428	13355	8342	6017
297	7323	9353	13316	13342	8307	5950
298	7192	9428	13235	13353	8256	5848
299	7060	9494	13195	13335	8231	5773
300	6927	9554	13187	13277	8251	5716
301	6804	9603	13232	13388	8251	5662
302	6640	9616	13270	13397	8224	5576
303	6501	9600	13330	13475	8208	5519
304	6371	9541	13408	13485	8200	5466
305	6236	9469	13479	13519	8179	5401
306	6101	9383	13557	13584	8095	5307
307	5970	9270	13633	13573	8055	5239

308	5802	9192	13711	13738	7987	5134
309	5675	9152	13784	13789	7889	5016
310	5562	9152	13832	13847	7858	4942
311	5405	9191	13861	13862	7810	4860
312	5245	9273	13858	13951	7728	4767
313	5076	9415	13806	13999	7654	4652
314	4912	9596	13712	14109	7621	4548
315	4772	9777	13594	14143	7568	4439
316	4604	9967	13428	14062	7525	4354
317	4427	10228	13207	14020	7506	4318
318	4229	10528	12911	13994	7477	4203
319	4032	10820	12588	14032	7395	4065
320	3894	11052	12337	13849	7345	3985
321	3712	11359	11962	13729	7340	3914
322	3554	11701	11544	13563	7298	3822
323	3329	12077	11031	13392	7238	3703
324	3152	12382	10566	13167	7210	3645
325	3039	12611	10226	12988	7185	3577
326	2844	12904	9672	12712	7139	3449
327	2666	13218	9129	12386	7106	3366
328	2492	13574	8535	12000	7070	3265
329	2324	13883	7943	11729	7032	3181
330	2208	14122	7517	11450	6973	3099
331	2032	14380	6927	10977	6990	3045
332	1882	14649	6350	10532	6997	2977
333	1696	14927	5687	10084	6985	2892
334	1561	15126	5183	9648	6967	2782
335	1441	15290	4765	9270	6926	2669
336	1304	15495	4270	8874	6879	2547
337	1201	15642	3833	8380	6819	2420
338	1056	15803	3330	7854	6842	2393
339	982	15928	2964	7450	6868	2357
340	895	16020	2674	7098	6874	2307
341	804	16067	2337	6636	6855	2229
342	733	16094	2044	6262	6784	2118
343	649	16117	1741	5831	6784	2094
344	587	16059	1487	5387	6819	2072
345	558	16046	1325	5121	6811	2036
346	514	16013	1157	4753	6829	1966
347	480	15879	999	4390	6824	1883
348	436	15770	836	4008	6789	1805
349	429	15624	723	3689	6710	1739
350	418	15409	617	3356	6679	1684
351	419	15229	542	3168	6690	1643
352	415	15015	463	2902	6628	1595
353	408	14728	394	2587	6596	1546
354	426	14455	337	2402	6564	1535
355	462	14154	301	2208	6504	1465
356	459	13857	256	1990	6462	1430
357	483	13501	218	1800	6358	1383
358	524	13128	193	1635	6334	1374

359	548	12756	161	1467	6244	1354
360	589	12323	144	1302	6170	1312
361	642	11965	135	1179	6106	1315
362	671	11553	117	1076	5989	1271
363	718	11192	103	990	5860	1232
364	784	10645	98	876	5675	1165
365	817	10230	78	818	5556	1106
366	864	9851	79	698	5484	1104
367	928	9408	71	653	5392	1080
368	935	8970	41	569	5211	1067
369	1014	8465	44	535	5075	1053
370	1061	8065	55	462	4969	1012
371	1105	7645	46	443	4860	988
372	1123	7265	24	389	4693	920
373	1184	6886	35	370	4598	971
374	1236	6399	33	350	4596	1024
375	1271	6032	25	332	4493	1018
376	1304	5684	26	294	4287	927
377	1342	5357	26	282	4099	867
378	1368	5014	21	260	3969	822
379	1393	4662	9	247	3860	791
380	1420	4285	11	229	3749	816
381	1441	4032	11	214	3606	786
382	1469	3783	19	215	3497	789
383	1476	3503	15	198	3381	770
384	1499	3210	13	199	3272	750
385	1514	2940	14	191	3160	712
386	1520	2752	12	179	3016	660
387	1526	2531	9	173	2922	651
388	1538	2352	11	173	2788	630
389	1536	2141	12	154	2717	613
390	1535	1955	12	165	2644	586
391	1531	1784	7	155	2561	569
392	1532	1656	11	144	2457	535
393	1524	1542	10	170	2383	530
394	1518	1396	10	148	2349	560
395	1511	1267	12	133	2271	548
396	1509	1169	10	131	2167	547
397	1496	1076	7	119	2067	508
398	1477	988	5	148	1997	473
399	1471	896	8	120	1898	434
400	1451	803	6	116	1792	422
401	1445	746	13	123	1738	405
402	1430	685	12	107	1695	365
403	1424	630	18	110	1642	370
404	1405	566	13	120	1554	344
405	1394	508	20	87	1503	333
406	1374	455	16	91	1467	324
407	1366	419	23	88	1416	319
408	1348	389	25	83	1328	304
409	1336	353	24	93	1256	276

410	1319	309	30	77	1216	275
411	1306	277	36	71	1182	262
412	1296	257	34	67	1099	245
413	1272	226	28	56	1006	185
414	1263	203	33	61	964	142
415	1244	183	33	61	918	127
416	1235	169	33	57	888	146
417	1218	157	33	57	851	152
418	1216	142	39	46	816	141
419	1209	128	40	92	741	103
420	1191	117	37	39	710	91
421	1177	110	39	50	716	126
422	1166	104	38	57	689	136
423	1156	100	37	48	634	132
424	1151	96	40	59	590	104
425	1139	79	31	46	582	94
426	1116	70	27	36	591	128
427	1097	58	13	22	517	93
428	1083	54	13	16	456	55
429	1071	45	4	21	418	26
430	1055	39	1	21	388	21
431	1045	37	4	9	389	46
432	1041	32	5	1	318	0
433	1034	27	8	5	326	49
434	1052	45	18	26	322	59
435	1041	44	22	21	272	47
436	1053	58	31	45	233	4
437	1057	63	42	47	184	0
438	1076	79	63	84	183	0
439	1045	60	36	65	170	0
440	1033	50	33	24	146	0
